# A Novel Mathematical Model Describing Adaptive Cellular Drug Metabolism and Toxicity in the Chemoimmune System

**DOI:** 10.1371/journal.pone.0115533

**Published:** 2015-02-20

**Authors:** Attila Tóth, Anna Brózik, Gergely Szakács, Balázs Sarkadi, Tamás Hegedüs

**Affiliations:** 1 MTA-SE Molecular Biophysics Research Group, Hungarian Academy of Sciences, Budapest, 1094, Hungary; 2 Department of Biophysics and Radiation Biology, Semmelweis University, Budapest, 1094, Hungary; 3 Institute of Enzymology, Research Centre for Natural Sciences, Hungarian Academy of Sciences, Budapest, 1113, Hungary; University of Cambridge, UNITED KINGDOM

## Abstract

Cells cope with the threat of xenobiotic stress by activating a complex molecular network that recognizes and eliminates chemically diverse toxic compounds. This “chemoimmune system” consists of cellular Phase I and Phase II metabolic enzymes, Phase 0 and Phase III ATP Binding Cassette (ABC) membrane transporters, and nuclear receptors regulating these components. In order to provide a systems biology characterization of the chemoimmune network, we designed a reaction kinetic model based on differential equations describing Phase 0–III participants and regulatory elements, and characterized cellular fitness to evaluate toxicity. In spite of the simplifications, the model recapitulates changes associated with acquired drug resistance and allows toxicity predictions under variable protein expression and xenobiotic exposure conditions. Our simulations suggest that multidrug ABC transporters at Phase 0 significantly facilitate the defense function of successive network members by lowering intracellular drug concentrations. The model was extended with a novel toxicity framework which opened the possibility of performing *in silico* cytotoxicity assays. The alterations of the *in silico* cytotoxicity curves show good agreement with *in vitro* cell killing experiments. The behavior of the simplified kinetic model suggests that it can serve as a basis for more complex models to efficiently predict xenobiotic and drug metabolism for human medical applications.

## Introduction

Living organisms are permanently exposed to potentially toxic xenobiotic compounds including environmental toxins and also drugs administered for therapeutic purposes. Although tissue barriers, such as the skin, the intestinal epithelia or the blood brain barrier limit the entry of xenobiotics into the body or a specific organ, individual cells have to cope with significant xenobiotic stress. The majority of the xenobiotics are detoxified through the canonical Phase I, II, and III pathways [1–5]. Phase I pathways include oxidative, reductive and hydrolysis reactions. The most prominent Phase I enzymes belong to the cytochrome P450 (CYP) family. CYPs (e.g. CYP3A4, CYP3A5, CYP2D6, CYP1A1, CYP1B1 and CYP2E1) recognize a wide range of chemicals as substrates, usually converting them into a more water soluble form [6]. The oxidized intermediates are further metabolized by the action of Phase II enzymes (e.g. UDP-glucuronosyltransferases—UGTs, glutathione S-transferases—GSTs), which neutralize Phase I products by conjugating them with small molecules [7]. Finally, conjugates are removed from the cells to avoid untoward accumulation. Phase III elimination is mostly linked to the activity of ABC (ATP Binding Cassette) transporters, including MRP1/ABCC1 and MRP2/ABCC2 [8, 9]. Additional ABC transporters (such as MDR1/ABCB1 and BCRP/ABCG2) can recognize unmodified xenobiotics and extrude them from the cell (or already from the cell membrane) in the so-called Phase 0 reaction, thus reducing the load on the entire metabolic process [4, 10].

The expression of phase 0-III enzymes and transporters is orchestrated by several nuclear receptors and transcription factors (e.g. NR1I2/PXR—pregnane X receptor, NR1I3/CAR—constitutive androstane receptor, and AhR—aryl hydrocarbon receptor) that recognize xenobiotics and often also their metabolites as ligands [1]. These regulatory processes converge to select for the most efficient set of proteins to protect the cell from the given xenobiotic. Based on the similarities of the cellular detoxification processes and the immune system (e.g. regulator and effector elements, differentiation of metabolites from xenobiotics (“self” vs. “nonself”, etc.), the complex network underlying cellular detoxication has been referred to as the “chemoimmune system” [4].

Therapeutic compounds are subject to cellular metabolism that influences both the ADME-Tox (absorption, distribution, metabolism, excretion, and toxicity) properties of drugs and also the drug-drug interactions [4, 9]. In many cases, metabolites may be more influential than parent xenobiotics. For example, it is estimated that three quarters of the carcinogens are activated from parent procarcinogenes by CYPs [11]. Conversely, bioactivation of prodrugs is desirable. Cyclophosphamide is activated by CYP to form its pharmacologically active phosphoramide mustard metabolite [12, 13]. Similarly, morphine-6-glucuronide produced by UGTs from morphine is a more potent analgesic than morphine itself [14]. Although the pharmacological relevance of the chemoimmune system is universally appreciated, little is known about the interplay of the individual enzymes or the kinetic parameters of the regulatory mechanisms. To fully appreciate the complexity of this elaborate system, dynamic interactions between the participating enzymes should be considered [15].

Mathematical models are promising possibilities for the investigation of such elaborate systems. On the other hand, xenobiotics interact with many regulators, enzymes and transporters, but the kinetic parameters describing these reactions are often unknown. Thus the formulation of widely applicable, general models is a serious challenge. Recent attempts to model the detoxifying cascade followed a single, well characterized xenobiotic or focused only on limited aspects of cellular detoxication. Stamatelos *et al*. created models describing the metabolism of arsenic compounds. While their toxicokinetic model [16] describes only Phase I-III reactions, the combined toxicokinetic-toxicodynamic model [17] includes also a simple transcriptional-translational feedback loop in the form of the Keap1–Nrf2 pathway playing a role in oxidative stress response regulation [18]. The electrophilic stress response model of Zhang and Andersen confines itself only to Phase II-III reactions, but contains more details about the Keap1–Nrf2 pathway [19]. Up to now, probably the model of Zhang *et al*. covers most aspects of cellular detoxication [20]. Beside Phase I-III reactions and the Keap1–Nrf2 pathway (the latter is described by not only feedback but also by feedforward loops) it contains regulatory circuits based also on the AhR nuclear receptor. However, since this model is geared towards mechanisms underlying overcompensation by the homeostatic control systems, especially hormetic response, and since the applied simplifications, it is suboptimal for more general modeling of the chemoimmune system.

To be able to study the role of each metabolite, enzyme and reaction involved in cellular detoxication in the context of this complex network, we generalized and extended the scheme proposed by Zhang *et al*. to create a more comprehensive model. We introduced ABC_0_ as a transporter modeling Phase 0 efflux transporters operating at pharmacological barriers. Furthermore, by making a clear distinction between xenobiotic metabolites and reactive species produced by the endogenous metabolic processes of the cell, we were able to analyze their relative importance. Using time course simulations we demonstrated our model’s ability to recapitulate elementary properties of the chemoimmune system. In addition, we introduced a novel framework to describe the effects of toxic species on cellular fitness. This opened the possibility for the calculation of *in silico* cytotoxicity curves resembling experimentally obtained results. Our results of *in silico* toxicity analysis are in agreement with RNA silencing experiments described in the literature [21, 22].

## Methods

### Generation of the model

The SBML (Level 2 Version 4) model format [23] was chosen for the implementation of the model. The model was created by using the graphical user interface of COPASI (version 4.8), the biochemical network simulator package [24] and custom Python scripts employing libSBML programming library (version 5.8.0) [25]. Time course simulations were carried out in COPASI, through its Python bindings. For numerical integration the deterministic (LSODA) solver was applied along with its default settings. Typically, the duration of the simulations was 60 hours with an interval size of 60 seconds. Initial conditions (here: concentrations) for time course simulations were steady state conditions corresponding to the given parameter set, and to zero extracellular xenobiotic concentration ([X_e_]). The initial conditions were calculated by running a preliminary, 30 day long time course simulation, sufficient for the system to reach steady state. Details of the model are provided as Supporting information.

### Biological and chemical details of the model

The chemoimmune network model is based on the scheme proposed by Zhang *et al*. [20], which describes the metabolism of xenobiotic X including the following reactions:

Phase I CYP oxidizes X to X’, Phase II GST conjugates GSH to X’ forming X”, which in turn is excreted from the cell by a membrane transporter (MRP). Transcription and translation of the participating enzymes are regulated by the AhR nuclear receptor and the Nrf2 transcription factor. The model contains three compartments: the extracellular space, the cytoplasm and the nucleus. In order to create a model tailored to the study of the chemoimmune system, this initial scheme was significantly modified (S1 Fig.). In brief, the following changes were introduced:

(1) ABC_0_, a Phase 0 transporter which is capable to eliminate the cytoplasmic form of X (X_c_) from the cell was introduced. (2) The transcriptional and translational regulation of ABC_0_ was added to the scheme. (3) Some parameters were altered to obtain biologically more realistic concentration ranges. For example the transcription rate constant of ABC_III_ (a general Phase III ABC transporter; formerly MRP) was reduced to decrease its level, while the transportation rate constant of X”_c_ was increased to keep its transportation rate roughly unaltered. For mathematical details of the model see S1 Model and S1–S4 Tables. (4) Reactive species originating from background reactions (X’_bc_) and its conjugated form (X”_bc_) were introduced (see Results for rationale). X’_bc_ and X”_bc_ were treated as respective xenobiotic metabolites X’_c_ and X”_c_. X’_c_ and X’_bc_ are substrates of GST, while X”_c_ and X”_bc_ are substrates of ABC_III_. To avoid extremely complex enzyme kinetics functions describing the metabolism of these two pairs of alternative substrates, instead of using conventional competitive inhibition formulae the following simplification was applied. In equations describing conjugation by GST and transport by ABC_III_, the total enzyme amount was divided according to the concentration ratios of the alternative substrates, and for each substrate this reduced amount of enzyme was used in the calculations. (5) X”_e_ (conjugated and extracellular product) and X”_be_ (conjugated and extracellular product from the background reactions) were introduced to be able to measure the amount of metabolites eliminated by ABC_III_. (6) A new aspect, a flexible description of cellular fitness was implemented using SBML parameters and events (S1 Text, S5–S6 Tables). (7) The ABC_III_ dimerization step was eliminated, since members of the ABCC family, participating in this Phase are full transporters. (8) MRP and AhR were renamed to ABC_III_ and NR, respectively to emphasize that they represent a general Phase III ABC transporter (e.g. MRP1, MRP2) and a general xenobiotic nuclear receptor, respectively. Other components related to the regulation by NR were also renamed.

### Calculation of cellular fitness

In order to simulate the impact of toxic species on cellular fitness, the model was extended with the variable *Fitness* (Fig. 5). To describe the toxicity of a drug or metabolite, its *Critical concentration* was defined as the threshold concentration, which must be exceeded to cause cellular damage when the given compound is assumed to form the sole toxic species in the cell. In addition to the inherent toxicity, the concentration and exposure time of toxic species also have to be considered for the assessment of cellular damage. To take the respective concentrations into account, the concentration of each toxic compound is divided by its *Critical concentration* and a nonlinear function of the sum of these fractions is defined as the *Chemical load. Chemical load* expresses chemical stress coming from all toxic species as a single value at any given time point.

The ability of the cells to recover from moderate damage caused by chemical stress is modeled by introducing a constant *Regeneration capacity.* According to the model, the cell can be in one of three states, defined by relations between *Chemical load, Regeneration capacity,* and *Fitness.* When *Chemical load* is lower than *Regeneration capacity,* repair mechanisms can compensate chemical stress, the cell may recover from previous damage. If *Fitness* is maximal, no regeneration occurs, since the cell is already in perfect shape. When *Fitness* is below its maximum, the cell undergoes regeneration to increase *Fitness.* When the *Chemical load* exceeds *Regeneration capacity,* repair mechanisms are insufficient to compensate chemical stress, the cell undergoes damage, *Fitness* is decreasing. The rate of regeneration and damage is the function of *Chemical load.* Thus, the exposure time is also taken into account in damage calculation. Regeneration and damage are described by the variables *Regeneration* and *Damage,* respectively.

Since cellular fitness is always the overall result of damage and regeneration, *Fitness* is calculated by adding the (scaled) positive values of *Regeneration* and the (scaled) nonpositive values of *Damage* to the maximal value of *Fitness.* At the start of the simulation experiment, *Fitness* has its maximal value (1), which represents the state of perfect health. Minimal *Fitness* (0) represents a state when the cell dies due to severe damage. Three characteristically different *Fitness* profiles are shown in S2 Fig. A more technical description of *Fitness* calculation is provided as Supporting information (S1 Text, S5–S6 Tables).

## Results

We have generated a new reaction kinetic model of the cellular chemoimmune network, based on the model proposed by Zhang *et al*. [20]. The Zhang-model was created to investigate hormesis, which was demonstrated through the function of participants of Phase I-III metabolism. Since our aim was to study the network as a whole system under a wider range of drug concentrations and various toxicity conditions, we have implemented several changes and additions.

**Fig 1:**
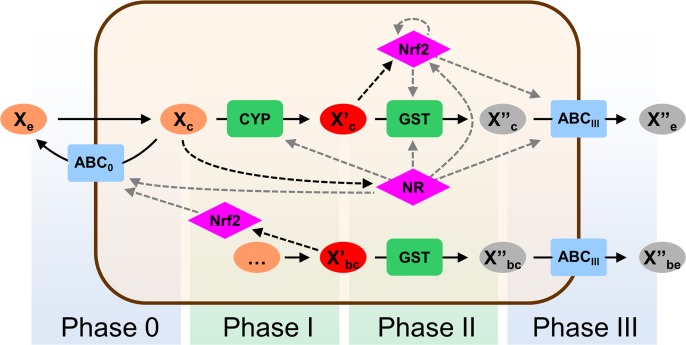
Simplified wiring diagram of the chemoimmune network model. Modeled interactions of a single xenobiotic (X) with Phase 0-III effector enzymes and regulators. Solid arrows represent transport through membranes or biochemical reactions. Dashed arrows denote regulation including multi-step transcriptional and translational regulation (gray) and more direct interaction (black), such as binding of a drug to nuclear receptors. ABC_0_ and ABC_III_ symbolize general Phase 0 and Phase III efflux transporters, respectively. CYP and GST represent a Phase I oxidase (a member of the cytochrome P450 superfamily) and a Phase II GSH transferase, respectively. NR symbolizes a general xenobiotic nuclear receptor, while Nrf2 denotes a specific transcription factor. (GST, ABC_III_ and Nrf2 are duplicated to increase clarity of the figure. Regulatory arrows are not duplicated.) Letters ‘c’ and ‘e’ indicate cytoplasmic and extracellular localization, respectively. X’_c_ is the CYP-oxidized cytoplasmic metabolite of X_c_. X”_c_ is the glutathione-conjugated form of X’_c_. X’_bc_ represents reactive species produced by normal cell metabolism. X’_bc_ is metabolized by the same pathway as X’_c_. Negative feedback loops are X_c_ → NR → CYP —| X_c_, X’_c_ (and X’_bc_) → Nrf2 → GST —| X’_c_ (and X’_bc_), X_c_ → NR → ABC_0_ —| X_c_, X_c_ → NR → Nrf2 → ABC_0_ —| X_c_, where → denotes activation and —| denotes inhibition. Feedforward loops are X_c_ → NR → GST —| X’_c_, X_c_ → NR → ABC_III_ —| X”_c_ (‘direct’ regulation) and X_c_ → NR → Nrf2 → GST —| X’_c_, X_c_ → NR → Nrf2 → ABC_III_ —| X”_c_ (‘indirect’ regulation). For the complete wiring diagram with all details see S1 Fig.

**Fig 2:**
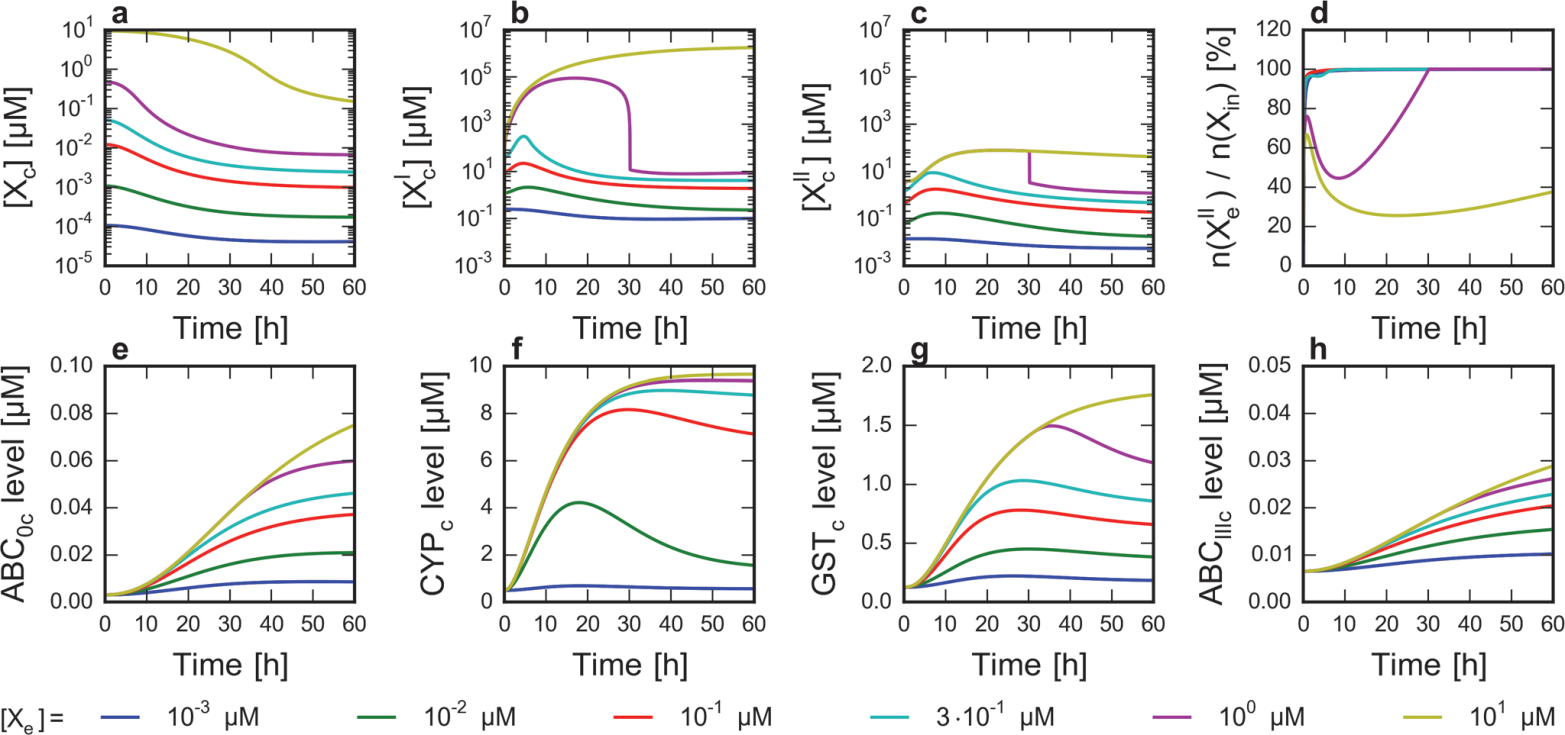
Effect of extracellular drug concentration on the level of network components. Six time course simulations were run from steady state as described in Methods. At t_0_ = 0 h the addition of drug was simulated by setting the extracellular drug concentration ([X_e_]) to a constant positive value between 1 nM and 10 μM. **a-c** Concentration profile of the cytoplasmic form of the drug ([X_c_]), its CYP-oxidized metabolite ([X’_c_]) and GST-conjugated form ([X”_c_]). **d** n(X”_e_)/n(X_in_) ratio, where n(X”_e_) is the amount of the ABC_III_-excreted extracellular form of X” and n(X_in_) is the amount of drug intake. (Details of drug intake calculation are provided in S1 Text.) **e-h** Concentration profile of four key enzymes (ABC_0_, CYP, GST and ABC_III_).

A simplified view of the chemoimmune model is shown in Fig. 1 (a more detailed description is provided in S1 Fig.). The model describes the interaction of a general xenobiotic compound (X) with a cell. The extracellular concentration of the xenobiotic ([X_e_]) is set to a constant value, based on the assumption that the extracellular space contains many orders of magnitude more X than the cell (at the end of the 60 hour long simulations in Fig. 2 the ratio of the cumulated drug intake and the total amount of drug in the extracellular space is in the range of 10^–7^), thus the amount of xenobiotic metabolized by the cell is negligible compared to the total available amount. The xenobiotic molecule enters the cell by diffusion through the plasma membrane. To model Phase 0 extrusion at “the gates” by plasma membrane ABC transporters such as ABCB1/MDR1, ABCC1/MRP1 or ABCG2/BCRP, we introduced ABC_0_, which eliminates X_c_, that is the unmetabolized, cytoplasmic form of the xenobiotic, thus limiting intracellular drug concentrations. X_c_ may also be converted to an oxidized metabolite (X’_c_) by CYP, or may bound to a nuclear receptor (forming XNR_c_), or may be distributed to the nucleus (X_n_) by diffusion. X’_c_ is conjugated by GST to form a more water-soluble metabolite (X”_c_), which is in turn eliminated by ABC_III_ (modeling Phase III efflux transporters) from the cell.

It should be noted that in the model of Zhang *et al*. X’_c_ represented not only the oxidized xenobiotic under investigation, but also reactive species (e.g. free radicals), produced by the basal metabolism of the cell. Since we focus on the effect of the xenobiotic and its metabolites, we introduced X’_bc_ and X”_bc_ to separately track background reactive species and their conjugated forms, respectively. This distinction allows the separate monitoring of the amount of xenobiotic metabolites and those produced by background processes. The intracellular forms of the xenobiotic (X_c_, X_n_, X’_c_, X”_c_) and the endogenous metabolites (X’_bc_ and X”_bc_) have distinct toxicity parameters, reflecting the respective toxicity of each metabolite.

Transcriptional and translational modulation of the effector transporters and metabolic enzymes in the cell is significantly affected by detailed regulatory circuits. In order to describe these processes the model contains a nuclear receptor, NR and the Nrf2 (NFE2L2) transcription factor [26]. NR provides a model for promiscuous nuclear receptors recognizing a wide range of drugs and xenobiotics. Since these regulatory elements interact with X_c_, X’_c_ and X’_bc_, the system contains multiple feedback and feedforward loops. The complete wiring diagram of the model is shown in S1 Fig.

Biochemical reactions of the modeled species are mathematically described by applying the laws of biochemical reaction kinetics. The resulting set of ordinary differential equations, their parameters and initial conditions together form the mathematical model. Since these differential equations are mostly nonlinear equations and cannot be solved analytically, numeric integration is required to solve them.

### The kinetic model recapitulates basic properties of the chemoimmune system

We performed simulations to investigate the basic behavior of the modeled network. First, the effect of extracellular xenobiotic concentrations on the expression levels of the network elements and the cellular concentrations of the produced metabolites were studied. Second, levels of various components were monitored as a function of the cellular uptake properties of the xenobiotic (by changing the passive diffusion rate). Third, the impact of changes in the affinities of a metabolic enzyme (GST) and a transporter (ABC_0_) to their substrates were followed.

As expected, increasing the extracellular xenobiotic concentration ([X_e_]) resulted in an increased cytoplasmic concentration of the xenobiotic ([X_c_]) and its oxidized ([X’_c_]) and conjugated ([X”_c_]) forms in the time course simulations (Fig. 2A-C). Following extracellular xenobiotic administration at 0 h, [X_c_] usually reaches a quasi-steady state level in seconds. Due to ABC_0_ activity, this level is always lower than the corresponding extracellular xenobiotic concentration. X_c_ triggers a regulatory response, which results in increasing ABC_0_ levels and monotonically decreasing [X_c_] on an hourly scale (Fig. 2A).

At relatively low xenobiotic doses, levels of X’_c_ and X”_c_ show a transient increase (at ca. 0–10 hours), before starting to decrease at 5 to 10 hours (Fig. 2B, C). This behavior is the result of multiple effects. First, X_c_ and X’_c_ upregulate the expression of transporters and metabolic enzymes (Fig. 2E-H). Initially, the CYP level grows faster than the GST level (compare Fig. 2F and G). As a result, the production rate of X’_c_ increases faster than its consumption rate, which results in the increase of [X’_c_]. Faster consumption of X_c_ by CYP at the same time leads to the faster decrease of [X_c_]. Decreasing X_c_ concentrations slow down the production of X’_c_ by decreasing the reaction rate of the CYP-catalyzed oxidation and by transcriptional and translational downregulation of CYP.

Interestingly, considering a wide range of extracellular xenobiotic concentrations, two characteristically different X’_c_ and X”_c_ concentration profiles can be distinguished (Fig. 2B, C). At lower xenobiotic doses, the transient concentration increase is moderate, as described above. When applying higher xenobiotic concentrations, X”_c_ levels reach a maximum, due to the saturation of GST (Fig. 2C). Since saturation of CYP happens only at higher substrate levels compared to GST, the oxidized xenobiotic accumulates, resulting very high X’_c_ concentrations (Fig. 2B).

Cellular accumulation of metabolites—an important factor in drug action—can also be inferred by calculating the ratio of the amount of the ABC_III_-excreted extracellular metabolite (n(X”_e_)) and the amount of cumulated xenobiotic intake (n(X_in_)) (Fig. 2D). At lower doses, the n(X”_e_)/n(X_in_) ratio is close to 100%, suggesting that most of the xenobiotic molecules are eliminated from the cell. Conversely, at higher xenobiotic concentrations this ratio drops significantly during the first hours of the simulation, which indicates severe metabolite accumulation.

It is important to note that the concentration of X_c_ is mostly in the nanomolar, while concentrations of X’_c_ and X”_c_ are in the micromolar range (Fig. 2A-C). This difference originates from the high capacity of CYP, producing X’_c_. Parameters of conjugation are based on experimental evidence, while parameters of oxidation were set by assuming CYP is not easily saturated. Since this enzyme catalyzes a practically irreversible reaction, and X’_c_ has no other way to be converted back to X_c_, in our model the consumed X_c_ can only be supplemented from other processes, which are the diffusion from the extracellular space (infinite source) and the nucleus, and dissociation from various complexes containing the unmetabolized form of X. Taking together, the model suggests that CYP activity can easily lead to X’_c_ concentrations orders of magnitude higher than the concentration of X_c_.

### Role of individual components in detoxication

**Fig 3:**
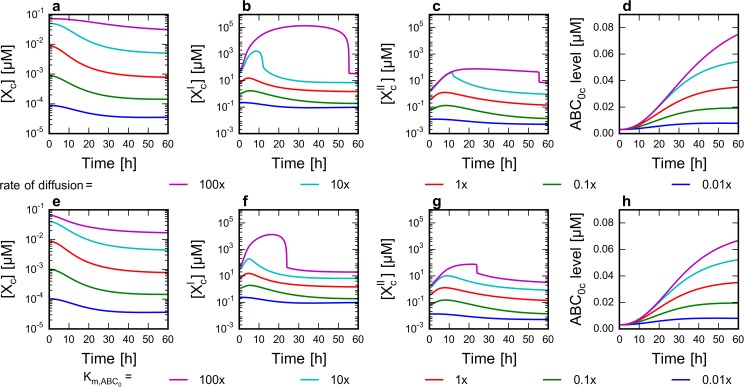
Effect of diffusion rate and ABC_0_ affinity to drug on the level of network components. Time course simulations were run from steady states belonging to different parameter sets as described in Methods. The extracellular drug concentration ([X_e_]) was set to 75 nM at t_0_ = 0 h. Parameter values are expressed as multiples of their default value (S3 Table). Concentration profile of X_c_, X’_c_, X”_c_ and the level of ABC_0_ were plotted. **a-d** Effect of diffusion rate through membranes. Five simulations were run by setting the diffusion rate constants to different values of four orders of magnitude. **e-h** Effect of ABC_0_ affinity to drug (K_m_). Five simulations were run by setting the Michaelis constant to different values encompassing four orders of magnitude.

The impact of individual components was studied by selectively altering the respective kinetic parameters. Increasing the passive diffusion rate of the xenobiotic through the membranes (which can reflect e.g. increased hydrophobicity of the xenobiotic) results in elevated cytoplasmic xenobiotic concentrations ([X_c_]) and—to a smaller extent—in elevated metabolite (X’_c_ and X”_c_) concentrations (Fig. 3A-D). When the diffusion rate is high, xenobiotic extrusion by ABC_0_ cannot significantly limit xenobiotic entry (Fig. 3A). Therefore, even though the concentration of X_c_ is much lower than the concentration of X’_c_, higher diffusion rates (or the presence of uptake transporters) potentially have severe adverse effects in the cell. Thus, similar to the case of high extracellular xenobiotic concentration (Fig. 2A, B), at high diffusion rates the saturation of GST leads to extremely high X’_c_ concentrations (Fig. 3A, B). These results highlight the relevance of xenobiotic diffusion rates in pharmacology, and also suggest that the incorporation of uptake transporters into the model could open further perspectives in understanding the kinetics of cellular xenobiotic metabolism [27]

**Fig 4:**
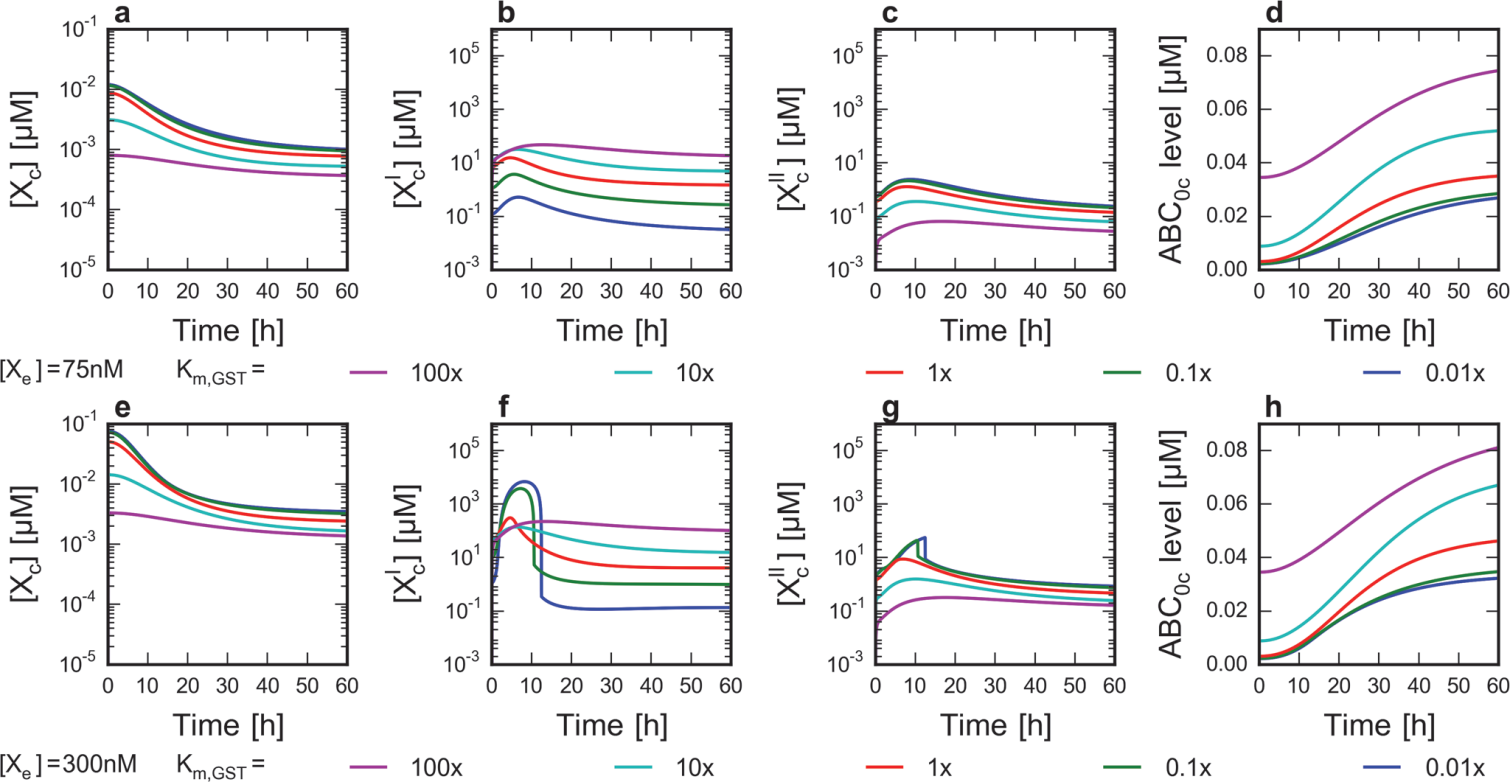
Effect of external drug concentration and GST affinity on the level of network components. Time course simulations were run from steady states belonging to different parameter sets as described in Methods. The Michaelis constants describing the affinity of GST to its substrates (X’_c_ and X’_bc_) were set to five different values encompassing four orders of magnitude. Parameter values are expressed as multiples of their default value (S3 Table). Concentration profile of X_c_, X’_c_, X”_c_ and the level of ABC_0_ were plotted. **a-d** The effect of GST affinity to its substrates (K_m_ values) at lower extracellular xenobiotic concentrations. [X_e_] was set to 75 nM at t_0_ = 0 h. **e-h** The effect of GST affinity to its substrates at higher extracellular xenobiotic concentrations. [X_e_] was set to 300 nM at t_0_ = 0 h.

We simulated the consequence of increased ABC_0_–mediated xenobiotic efflux by decreasing the apparent Michaelis constant (K_m;_ Fig. 3E-H). In case of moderate substrate concentrations, lower K_m_ values result in increased efflux, which leads to lower [X_c_], and consequently also to reduced metabolite (X’_c_ and X”_c_) concentrations. As expected, the increased diffusion rate can be compensated by an increase in the affinity of ABC_0_ (S3 Fig.). Similarly, by lowering the K_m_ of GST, X’_c_ concentrations decrease (Fig. 4B), while X”_c_ levels increase due to the higher production rate (Fig. 4C).

Interestingly, our model predicts that reactive species produced by normal cell metabolism (X’_bc_) may play an important role in preconditioning the chemoimmune system. Decreased affinity of GST to X’_bc_ (increased K_m_) results in elevated baseline concentrations of X’_bc_, which in turn leads to increased baseline ABC_0_ levels (since X’_bc_ upregulates ABC_0_ through the Nrf2 pathway), even in the absence of extracellular xenobiotics. (Compare basal ABC_0_ levels at t = 0 h on Fig. 4H which are steady state values calculated before simulating the addition of the xenobiotic to the system.) The baseline ABC_0_ level has a twofold significance. As expected, ABC_0_ function results in lower X_c_ levels (Fig. 4A, E). In addition, when higher xenobiotic doses are applied, increased baseline ABC_0_ activity protects the cells against initial [X’_c_] peaks (Fig. 4F). Thus, the presence of reactive species (e.g. free radicals) increases the initial detoxication performance of the cell. This feature of the system can be viewed also as a hallmark of hormesis: moderate stress may be beneficial for the cell to react faster during the need of chemodefense [28].

### Model of cellular fitness to study toxicity

**Fig 5:**
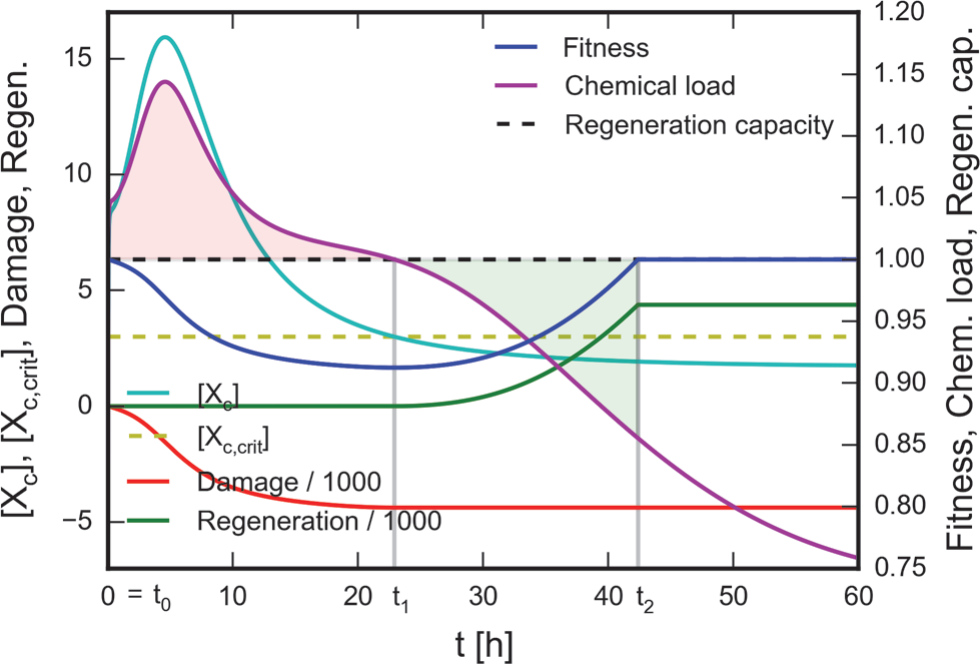
Modeling cellular fitness to study cytotoxic effects. Calculation of cellular fitness by assuming a single toxic compound, X. Cytoplasmic concentration of the drug ([X_c_]) was calculated using the time course simulation described in Methods. *Critical concentration* of X_c_ ([X_c,crit_], dashed yellow line) was defined as the threshold concentration, which must be exceeded to cause cellular damage. *Chemical load* is defined as a nonlinear function of the [X_c_]/[X_c,crit_] ratio (magenta curve). The cell is assumed to have a constant *Regeneration capacity* (dashed black line). When *Chemical load* exceeds *Regeneration capacity (t0 < t < t1)*, the cell undergoes damage. Cellular damage is represented by the *Damage* variable (red curve), which has nonpositive values proportional to the light red shaded area. When *Chemical load* is below *Regeneration capacity* and *Fitness* is below of its maximal value (*t1 < t < t2*), the cell undergoes regeneration. Regeneration is represented by the *Regeneration* variable (green curve), which has nonnegative values proportional to the green shaded area. When *Chemical load* is below *Regeneration capacity* but *Fitness* is maximal (*t2 < t*), nor damage, neither regeneration occurs. *Fitness* (blue curve) is calculated by adding the (scaled) nonnegative values of *Regeneration* and the (scaled) nonpositive values of *Damage* to the maximal value of *Fitness*. See text for details.

In addition to describing changes in the concentration of intracellular xenobiotic forms, we wished to model the cytotoxic effect of the compounds. To this end we extended the model with a versatile description of the impact of toxic species on cellular fitness (Fig. 5), described by the variable *Fitness* (blue curve). This is a general description which does not contain any assumption about the mechanism of toxicity, and should be considered as an approximation rather than a detailed representation of toxic effects.

Typical toxicity studies use only single parameters (e.g. EC_50_, LD_50_), and are not informative regarding the toxicity of the various intracellular drug forms. In our model, we have assigned a toxicity parameter to each intracellular form of the xenobiotic, and indirectly calculated an overall EC_50_-like value, allowing the simulation of toxic effects of drugs with different ‘toxicity profiles’. See Methods for implementation details of *Fitness*‘ calculation.

### *In silico* cytotoxicity curves

**Fig 6:**
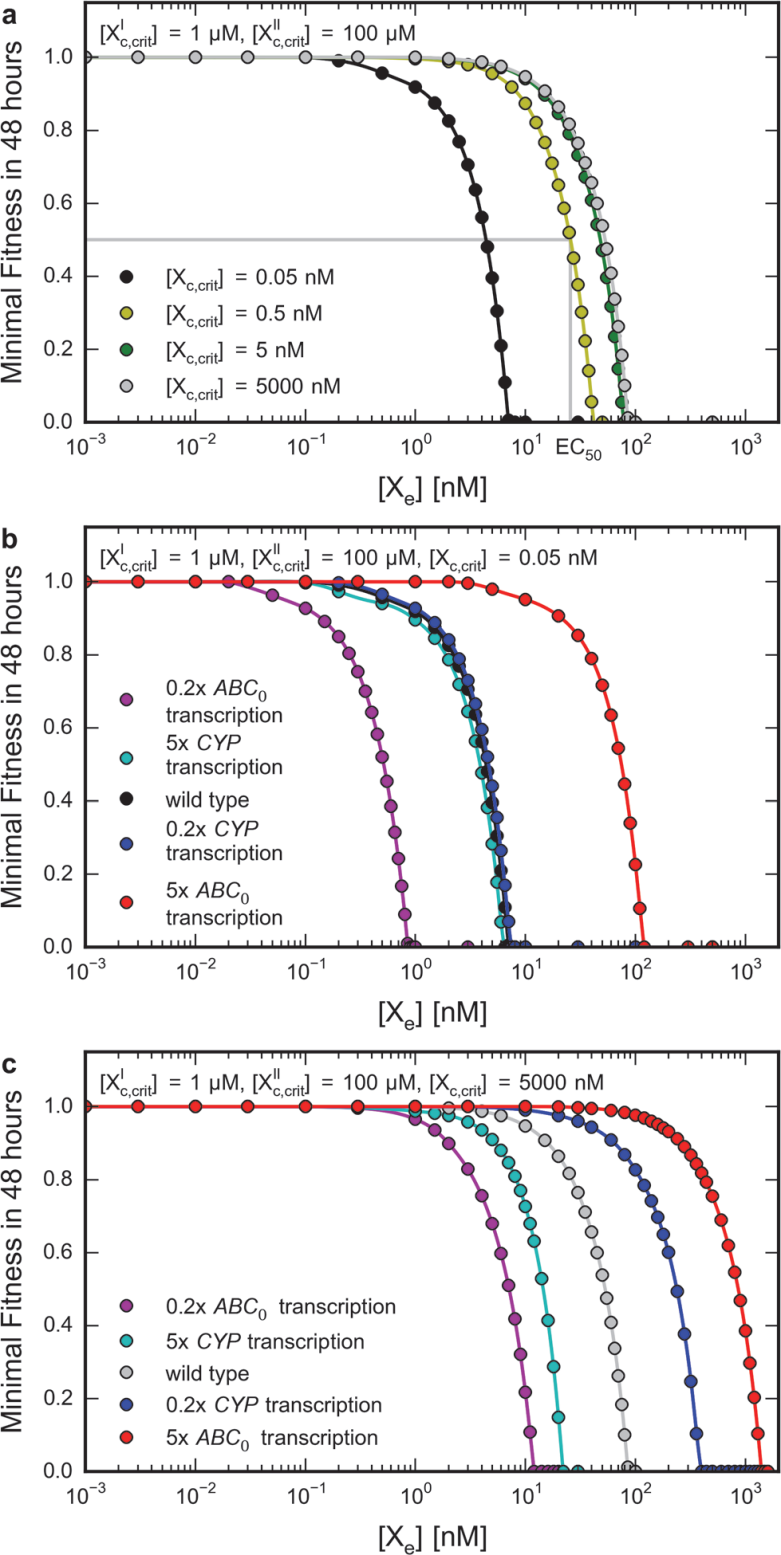
*In silico* cytotoxicity curves reveal impact of drug’s toxicity profile and protein level on survival. Time course simulations were run for up to 48 hours from steady states belonging to different parameter sets and extracellular drug concentrations ([X_e_], set at t_0_ = 0 h) as described in Methods. The minimal *Fitness* values reached in simulations were plotted against [X_e_] (colored circles; connected by interpolation curves—see S1 Text). Critical concentrations of X’_c_ and X”_c_ are constant on all panels with values 1 μM and 100 μM, respectively. Critical concentration of X_c_ ([X_c,crit_]) is indicated on the panels. **a** Impact of the toxicity of the unmetabolized form of the drug ([X_c,crit_]) on *in silico* cytotoxicity. *In silico* cytotoxicity curves were plotted for [X_c,crit_] values from 0.05 nM to 5 μM. The EC_50_ value is indicated for [X_c,crit_] = 0.5 nM. **b** Impact of transporter and oxidase levels on *in silico* cytotoxicity when X_c_ is more toxic than X’_c_ ([X_c,crit_] = 0.05 nM). **c** Impact of transporter and oxidase levels on *in silico* cytotoxicity when X_c_ is less toxic than X’_c_ ([X_c,crit_] = 5 μM). In panels **b** and **c** *in silico* cytotoxicity curves were calculated after setting the transcription rates of ABC_0_ or CYP 0.2 or 5 times of its original value (S3 Table) to model lower or higher expression levels.

In order to test one of the possible practical applications of the kinetic model, we aimed to calculate drug cytotoxicity curves. The analogous diagrams in the model are *Minimal Fitness vs [Xe]* curves, generated by running multiple time course simulations, by using increasing extracellular xenobiotic concentrations and measuring the minimal *Fitness* values in 48 hours (the length of a typical *in vivo* cytotoxicity test) (Fig. 6). *Minimal Fitness* is used instead of the *Fitness* value belonging to the endpoint of the simulation, as the final value reflects the actual state of the cell and does not include information on decreased metabolism (e.g. cell division), which is measured in *in vivo* experiments. Therefore, similar to an experimental cytotoxicity curve, an *in silico* cytotoxicity curve also allows the deduction of the EC_50_ value: it can be defined as the external xenobiotic concentration which causes 50% decrease of *Fitness* in the time course of 48 hours.

An important difference between experimentally determined and *in silico* cytotoxicity curves is that the former is calculated by investigating a cell population, and the latter is calculated by deterministic modeling of a hypothetical single cell. In the theoretical case, minimal Fitness values between 1 and 0 can be interpreted like partial growth inhibition, and zero Fitness as cell death.

First, we investigated the impact of the toxicity of the unmetabolized form ([X_c,crit_]) on the *in silico* cytotoxicity curve (Fig. 6A). Assuming that X’_c_ is more toxic than X”_c_ (this is the typical case) the critical concentrations of X’_c_ and X”_c_ were set to constant values of 1 μM and 100 μM, respectively. The critical concentration of X_c_ was varied between 0.05 nM and 5 μM. When [X_c,crit_] = 0.05 nM, and X_c_ is more toxic than X’_c_, the corresponding curve was shifted to lower concentrations (EC_50_ = 4.38 nM) from the curve with [X_c,crit_] = 0.5 nM (EC_50_ = 25.72 nM). Contrary, when [X_c,crit_] = 5 μM, and X_c_ is less toxic than X’_c_, the corresponding curve was shifted to higher concentrations (EC_50_ = 47.97 nM). The curves with [X_c,crit_] = 5 nM and 5 μM are close to each other, indicating a maximal EC_50_ threshold close to [X_e_] = 53 nM. The existence of the threshold can be explained as follows: when [X_e_] is above this threshold, [X’_c_] exceeds [X’_c,crit_] during a long period of time, resulting in cell death.

Next, we investigated the influence of altered levels of ABC_0_ and CYP in two cases of different toxicity conditions. In the first case the drug is more toxic than its oxidized metabolite. This is a typical scenario, e.g. most docetaxel metabolites are more than 500 times less toxic than the drug itself [29]. In the opposite case the drug is less toxic than its oxidized metabolite. Among the important examples are antitumor prodrugs like cyclophosphamide [12]. As expected, when the ABC_0_ substrate X_c_ is more toxic than X’_c_, changes in ABC_0_ levels lead to much more pronounced effects than changes in CYP expression levels (Fig. 6B). Decrease of ABC_0_ level results in lower EC_50_ values, while an increase of ABC_0_ level results in higher EC_50_ values. However, when X_c_ is less toxic than X’_c_ (Fig. 6C), the effect of CYP modulation is more significant. Modulation of ABC_0_ is still important, since it indirectly influences the X’_c_ level. In this case both decrease of ABC_0_ and increase of CYP levels results in lower EC_50_ values, while increase of ABC_0_ and decrease of CYP level results in higher EC_50_ values. These results are in agreement with RNA silencing experiments, where cytotoxicity to doxorubicin increased after silencing of *ABCG2* [22], whereas cytotoxicity to cyclophosphamide decreased after the silencing of *CYP3A4* [21].

## Discussion

In order to analyze the cellular chemoimmune network from a systems biology perspective, we have developed a detailed reaction kinetic model. Our model is the first to describe the metabolism of a single, general xenobiotic or drug covering all aspects of cellular detoxication from Phase 0 to Phase III and reflecting xenobiotic dependent transcriptional regulation. In addition to effectors (transporters and metabolic enzymes converting chemicals) the kinetic model also contains regulatory elements, including a general xenobiotic sensing nuclear receptor, the Keap1–Nrf2 oxidative stress response pathway, and sophisticated transcriptional and translational regulatory circuits. These pathways form multiple feedback and feedforward loops, through which the xenobiotic and its oxidized metabolite regulate their own transport and metabolism. Of course, any of these regulatory mechanisms are independently switchable by setting the appropriate reaction kinetic parameters. This way modeling the metabolism of xenobiotics e.g. without Phase 0 excretion is also possible.

The chemoimmune model recapitulates basic properties of the chemoimmune system. Increasing extracellular xenobiotic concentrations result in increased cytoplasmic concentrations of the xenobiotic metabolites, which in turn lead to the upregulation of transporters and metabolic enzymes through transcriptional and translational feedback and feedforward loops (Fig. 2). The cytoplasmic concentration of the unmetabolized xenobiotic is jointly affected by its diffusion rate, Phase 0 transporter activity and consumption by metabolic processes (Fig. 3). We find that increased affinity (decreased K_m_) of a Phase 0 xenobiotic transporter for the xenobiotic can counteract increased xenobiotic influx (increased rate of diffusion) (S3 Fig.).

In various time course simulations the concentrations of the xenobiotic, its metabolites, metabolic enzymes and efflux transporters were followed and relevant scenarios corresponding to particular drug effects were studied. Simulations clearly indicate that the metabolites can reach much higher concentrations than the external xenobiotic concentration especially when an enzyme, which forms a bottleneck in the metabolic pathway is saturated, thus leading to an extremely elevated concentration of its substrate (Fig. 2). An example of this feature in our model is GST, which conjugates GSH to the oxidized form of the xenobiotic. Since the oxidized form neither can be reverted back to reduced form, nor can be removed by other pathways, saturation of GST leads to an extreme increase in the level of the oxidized form (Fig. 2B, D). In a real cell, the oxidized form may be cleared by pathways other than GSH conjugation. On the contrary, the unmetabolized form of the xenobiotic cannot be concentrated in the cell, since even in the case of limited oxidase (CYP) action, it still can be cleared at Phase 0 due to ABC transporter action. The model suggests that the role of Phase 0 exporters extends beyond limiting the cellular entry of toxic molecules to prevent the buildup of extreme metabolite concentrations and the saturation of metabolic enzymes.

Interestingly, saturation of metabolic enzymes is also prevented by reactive species produced by endogenous metabolic processes of the cell. Our simulations suggest that the increased levels of reactive species produced by normal cell metabolism prevent extreme metabolite peaks (Fig. 4F) by elevating basal enzyme expression levels (Fig. 4H). Thus, a low level exposure to toxic molecules can prepare the system for a massive attack of xenobiotics. This characteristic of the chemoimmune system resembles the phenomenon of active immunization in the context of the immune system, making the analogy between the immune and chemoimmune systems even more fascinating.

By means of the newly developed toxicity framework, the model became suitable to predict toxicity parameters of *in silico* cytotoxicity assays (Fig. 6). The toxicity module—despite being simplified—is able to qualitatively reproduce the results of cell killing experiments [21, 22]. An important limitation of our model in this regard is that in order to provide predictive systemic effects, the toxicological properties of the modeled drug metabolites have to be independently estimated. Still, from relevant experiments these values can be reinserted into the model to study the effects of variable cellular parameters.

An important medical aspect is the involvement of the chemoimmune network and more specifically multidrug ABC transporters in cancer drug resistance, a major cause of the failure of chemotherapy in malignant diseases. Potent inhibitors against ABCB1 have been developed to overcome cancer multidrug resistance (MDR), but the therapeutic applications were unsuccessful in major clinical trials [30]. These results underline the importance of ABC transporter analysis in the context of the whole chemoimmune network [31, 32]. By providing a system level description of this network, the chemoimmune model provides powerful means for the comprehension of side-effects caused by ABC transporter inhibition, and may serve as a valuable aid in the development of novel therapeutic strategies.

In summary, the chemoimmune model describes cellular xenobiotic metabolism well at the level of the applied simplification. In its present form, it is a capable tool for the investigation of dynamic interactions between components of the chemoimmune system and for the assessment of toxic effects on cellular viability. Also, it can serve as a basis of forthcoming, more advanced models. Introduction of xenobiotic uptake transporters (e.g. members of the SLC family) with saturable properties as compared to passive xenobiotic diffusion, implementation of further xenobiotic sensing nuclear receptors or further metabolic enzymes and transporters may lead to more realistic models suitable not only for qualitative, but also for quantitative predictions.

## Supporting Information

S1 Fig Detailed representation of the model of the chemodefense network. Wiring diagram of the chemodefense network represented as an SBGN [33] diagram.
(PDF)

S2 Fig Characteristic ‘Fitness profiles’ for three different external drug concentrations. Three time course simulations were ran from steady state as described in Methods after setting different extracellular drug concentrations ([X_e_]) at t_0_ = 0 h. Critical concentration of X_c_, X’_c_ and X”_c_ was set to 5 nM, 5 μM and 5 mM, respectively (S3 Table). Concentration of X’_c_ (solid curves), and *Fitness* (dashed curves) were plotted. Simulating drug administration by setting relatively low external drug concentration (green curves) results only in transient *Fitness* decrease, since decreasing concentration of toxic compounds allows regeneration. Moderate drug concentration (blue curves) leads to monotonic *Fitness* decrease to an intermediate level till the end of the simulation experiment. Considering that typical cytotoxicity tests are run up to 24 or 48 hours, the observed behavior can be interpreted as the sign of cytostasis or partial growth inhibition. When applying relatively high drug concentration (red curves), *Fitness* decreases to zero before the end of the experiment, which signs the death of the cell. When the cell dies, simulation is interrupted.
(PDF)

S3 Fig Simultaneous alteration of diffusion rate and ABC_0_ affinity to drug can compensate each other’s effect. Five time course simulations were run from steady states belonging to different parameter sets as described in Methods. The Michaelis constant of ABC_0_ and the diffusion rate constants were altered simultaneously. The extracellular drug concentration ([X_e_]) was set to 75 nM at t_0_ = 0 h. Similarity of concentration profiles indicate that opposing effects affecting xenobiotic transport through membranes can compensate each other. Parameter values are expressed as multiples of their default value (S3 Table) **a-c** Concentration profile of the cytoplasmic form of the drug ([X_c_]), its CYP-oxidezed metabolite ([X’_c_]) and the GST-conjugated form of the latter ([X”_c_]). **d** Concentration profile of ABC_0_.
(PDF)

S1 Model Mathematical model. The model is provided as a separate SBML (Level 2 Version 4) format [23] file.
(XML)

S1 Table Equations of the model. For parameters and initial values see S2 and S4 Tables, respectively. Parameter names may contain colons, slashes, and parentheses as shown in S3 Table. Multiplication and division is always indicated by centered dots and fractions, respectively. Equations of fitness calculation are shown in S5 Table.
(PDF)

S2 Table Parameters of the model. SBML IDs, names, and default (wild type) values of ‘fixed’ type model parameters. For corresponding equations see S1 Table.
(XLS)

S3 Table Parameters changed in simulation experiments. Parameters changed (compared to their default values shown in S2 Table) in simulation experiments.
(PDF)

S4 Table Initial values of the model. SBML IDs, species’ names, species’ compartments, and initial values. For corresponding equations see S1 Table.
(XLS)

S5 Table Mathematical details of cellular fitness calculation. SBML parameter types: F: fixed, A: assignment, O: ODE, B: Boolean (fixed with values 0 and 1, set by events), E: fixed, set by events. Min. and Max. values in parentheses indicate the actual values of minimums and maximums, respectively. For events see S6 Table.
(PDF)

S6 Table SBML events used in cellular fitness calculation. For variable names see S5 Table.
(PDF)

S1 Text Supplementary methods. Details of cellular fitness, in silico cytotoxicity curve, and drug intake calculation.
(PDF)
