# Supplementary figures and images for: A Novel Mathematical Model Describing Adaptive Cellular Drug Metabolism and Toxicity in the Chemoimmune System
Source: PLoS One. 2015 Feb 20;10(2):e0115533. doi: 10.1371/journal.pone.0115533 (PMC4338831; doi:10.1371/journal.pone.0115533)

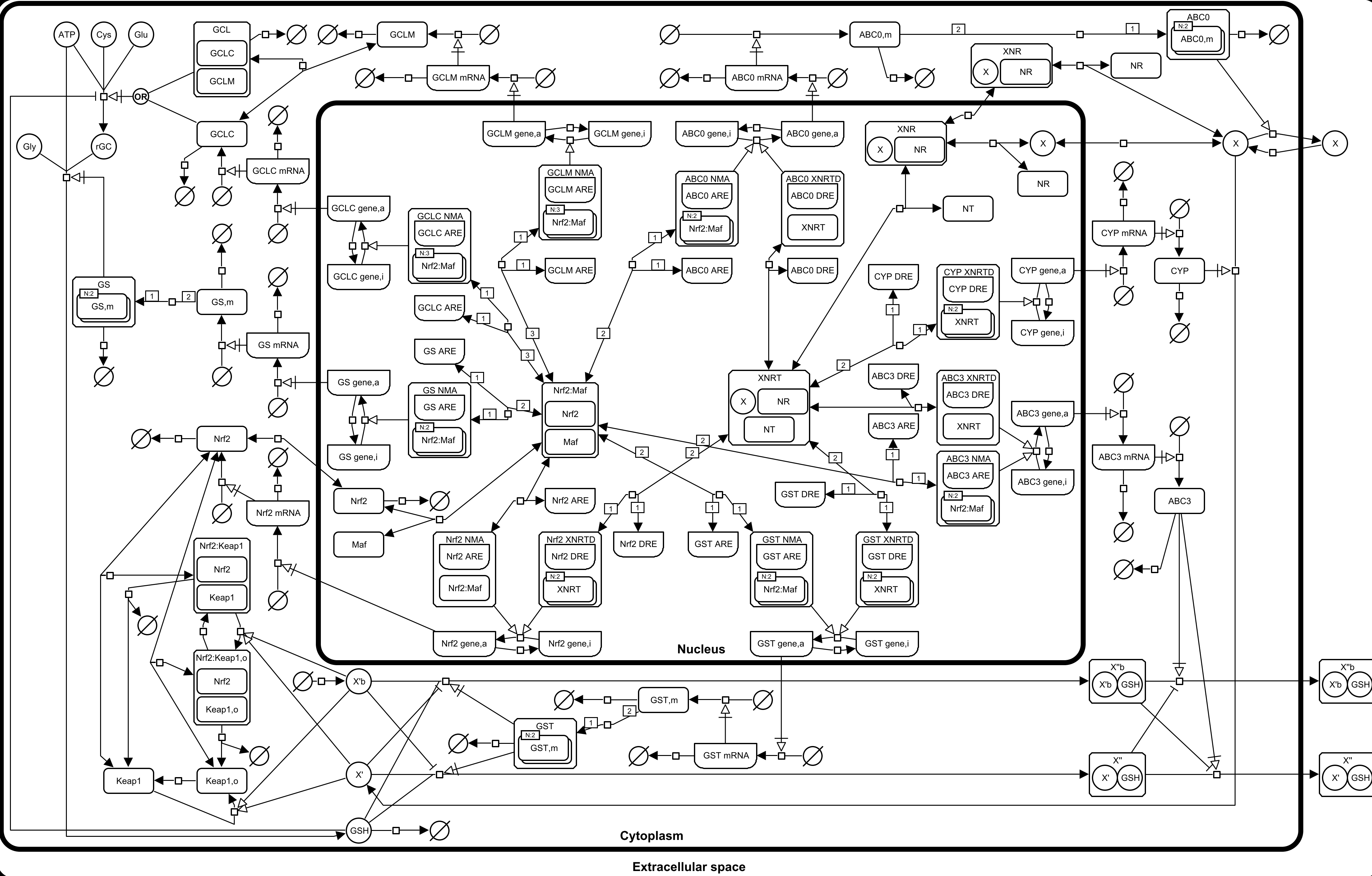

Supplement: S1 Fig — Wiring diagram of the chemodefense network represented as an SBGN [33] diagram. (PDF) [file pone.0115533.s001.pdf]

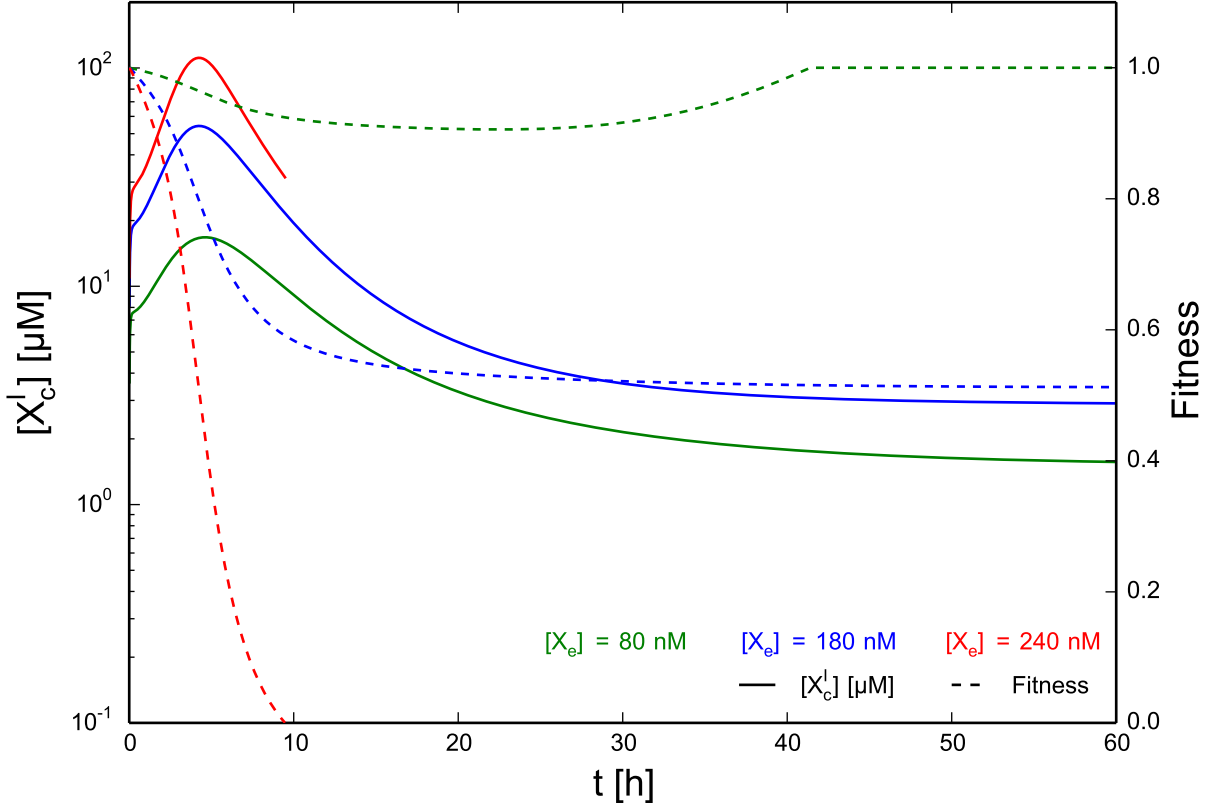

Supplement: S2 Fig — Three time course simulations were ran from steady state as described in Methods after setting different extracellular drug concentrations ([Xe]) at t0 = 0 h. Critical concentration of Xc, X’c and X”c was set to 5 nM, 5 μM and 5 mM, respectively (S3 Table). Concentration of X’c (solid curves), and Fitness (dashed curves) were plotted. Simulating drug administration by setting relatively low external drug concentration (green curves) results only in transient Fitness decrease, since decreasing concentration of toxic compounds allows regeneration. Moderate drug concentration (blue curves) leads to monotonic Fitness decrease to an intermediate level till the end of the simulation experiment. Considering that typical cytotoxicity tests are run up to 24 or 48 hours, the observed behavior can be interpreted as the sign of cytostasis or partial growth inhibition. When applying relatively high drug concentration (red curves), Fitness decreases to zero before the end of the experiment, which signs the death of the cell. When the cell dies, simulation is interrupted. (PDF) [file pone.0115533.s002.pdf]

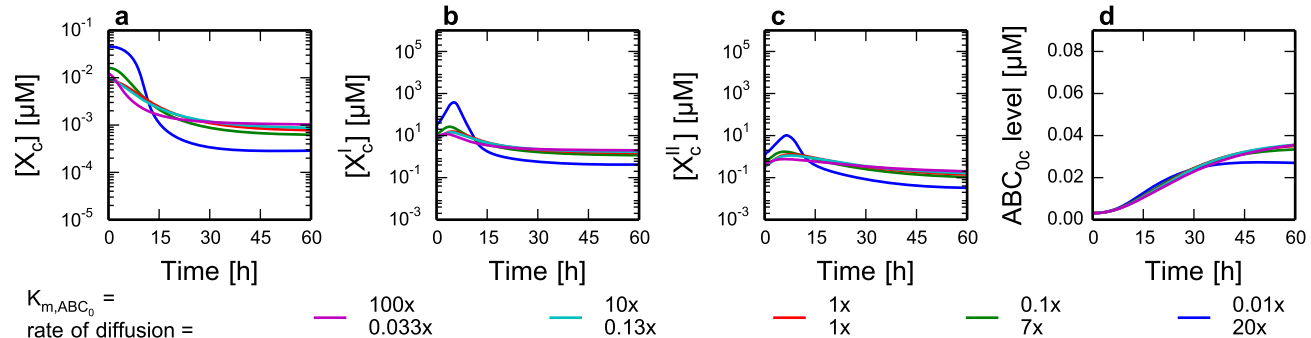

Supplement: S3 Fig — Five time course simulations were run from steady states belonging to different parameter sets as described in Methods. The Michaelis constant of ABC0 and the diffusion rate constants were altered simultaneously. The extracellular drug concentration ([Xe]) was set to 75 nM at t0 = 0 h. Similarity of concentration profiles indicate that opposing effects affecting xenobiotic transport through membranes can compensate each other. Parameter values are expressed as multiples of their default value (S3 Table) a-c Concentration profile of the cytoplasmic form of the drug ([Xc]), its CYP-oxidezed metabolite ([X’c]) and the GST-conjugated form of the latter ([X”c]). d Concentration profile of ABC0. (PDF) [file pone.0115533.s003.pdf]
