# Supplementary material for: A Novel Mathematical Model Describing Adaptive Cellular Drug Metabolism and Toxicity in the Chemoimmune System
Source: PLoS One. 2015 Feb 20;10(2):e0115533. doi: 10.1371/journal.pone.0115533 (PMC4338831; doi:10.1371/journal.pone.0115533)
Supplement: S1 Table — For parameters and initial values see S2 and S4 Tables, respectively. Parameter names may contain colons, slashes, and parentheses as shown in S3 Table. Multiplication and division is always indicated by centered dots and fractions, respectively. Equations of fitness calculation are shown in S5 Table. (PDF) [file pone.0115533.s005.pdf]

$$\frac{d([ABC0,m\_c] \cdot V\_Cytosol)}{dt} = -V\_Cytosol \cdot (k\_d\_ABC0,m\_c \cdot [ABC0,m\_c])$$
$$-2 \cdot V\_Cytosol \cdot ((\text{"k\_as\_ABC0,m\_c\_(ABC0,m\_c)"} \cdot [ABC0,m\_c] \cdot [ABC0,m\_c] - k\_ds\_ABC0\_c \cdot [ABC0\_c]))$$
$$+V\_Cytosol \cdot (k\_tl\_ABC0\_mRNA\_c \cdot [ABC0\_mRNA\_c])$$

$$\frac{d([ABC0\_c] \cdot V\_Cytosol)}{dt} = -V\_Cytosol \cdot (k\_d\_ABC0\_c \cdot [ABC0\_c])$$
$$+V\_Cytosol \cdot ((\text{"k\_as\_ABC0,m\_c\_(ABC0,m\_c)"} \cdot [ABC0,m\_c] \cdot [ABC0,m\_c] - k\_ds\_ABC0\_c \cdot [ABC0\_c]))$$

$$\frac{d([ABC0\_mRNA\_c] \cdot V\_Cytosol)}{dt} = +V\_Cytosol \cdot \left(\frac{k\_ts\_ABC0\_gene,a\_n \cdot [ABC0\_gene,a\_n] \cdot \frac{1}{3} \cdot V\_Nucleus}{V\_Cytosol}\right)$$
$$-V\_Cytosol \cdot (k\_d\_ABC0\_mRNA\_c \cdot [ABC0\_mRNA\_c])$$

$$\frac{d([ABC3\_c] \cdot V\_Cytosol)}{dt} = +V\_Cytosol \cdot (k\_tl\_ABC3\_mRNA\_c \cdot [ABC3\_mRNA\_c])$$
$$-V\_Cytosol \cdot (k\_d\_ABC3\_c \cdot [ABC3\_c])$$

$$\frac{d([ABC3\_mRNA\_c] \cdot V\_Cytosol)}{dt} = +V\_Cytosol \cdot \left(\frac{k\_ts\_ABC3\_gene,a\_n \cdot [ABC3\_gene,a\_n] \cdot \frac{1}{3} \cdot V\_Nucleus}{V\_Cytosol}\right)$$
$$-V\_Cytosol \cdot (k\_d\_ABC3\_mRNA\_c \cdot [ABC3\_mRNA\_c])$$

$$\frac{d([CYP\_c] \cdot V\_Cytosol)}{dt} = -V\_Cytosol \cdot (k\_d\_CYP\_c \cdot [CYP\_c])$$
$$+V\_Cytosol \cdot (k\_tl\_CYP\_mRNA\_c \cdot [CYP\_mRNA\_c])$$

$$\frac{d([CYP\_mRNA\_c] \cdot V\_Cytosol)}{dt} = +V\_Cytosol \cdot \left(\frac{k\_ts\_CYP\_gene,a\_n \cdot [CYP\_gene,a\_n] \cdot 5 \cdot V\_Nucleus}{V\_Cytosol}\right)$$
$$-V\_Cytosol \cdot (k\_d\_CYP\_mRNA\_c \cdot [CYP\_mRNA\_c])$$

$$\frac{d([GCL\_c] \cdot V\_Cytosol)}{dt} = -V\_Cytosol \cdot (k\_d\_GCL\_c \cdot [GCL\_c])$$
$$+V\_Cytosol \cdot ((\text{"k\_as\_GCLC\_c\_(GCLM\_c)"} \cdot [GCLM\_c] \cdot [GCLC\_c] - k\_ds\_GCL\_c \cdot [GCL\_c]))$$

$$\frac{d([GCLC\_c] \cdot V\_Cytosol)}{dt} = -V\_Cytosol \cdot ((\text{"k\_as\_GCLC\_c\_(GCLM\_c)"} \cdot [GCLM\_c] \cdot [GCLC\_c] - k\_ds\_GCL\_c \cdot [GCL\_c]))$$
$$-V\_Cytosol \cdot (k\_d\_GCLC\_c \cdot [GCLC\_c])$$
$$+V\_Cytosol \cdot (k\_tl\_GCLC\_mRNA\_c \cdot [GCLC\_mRNA\_c])$$

$$\frac{\mathrm{d}([\text{GCLC\_mRNA\_c}] \cdot V\text{\_Cytosol})}{\mathrm{d}t} = +V\text{\_Cytosol} \cdot \left(\frac{\text{k\_ts\_GCLC\_gene,a\_n} \cdot [\text{GCLC\_gene,a\_n}] \cdot \frac{1}{3} \cdot V\text{\_Nucleus}}{V\text{\_Cytosol}}\right)$$

$$-V\text{\_Cytosol} \cdot (\text{k\_d\_GCLC\_mRNA\_c} \cdot [\text{GCLC\_mRNA\_c}])$$

$$\frac{\mathrm{d}([\text{GCLM\_c}] \cdot V\text{\_Cytosol})}{\mathrm{d}t} = -V\text{\_Cytosol} \cdot ((\text{”k\_as\_GCLC\_c\_(GCLM\_c)”} \cdot [\text{GCLM\_c}] \cdot [\text{GCLC\_c}] - \text{k\_ds\_GCL\_c} \cdot [\text{GCL\_c}]))$$

$$-V\text{\_Cytosol} \cdot (\text{k\_d\_GCLM\_c} \cdot [\text{GCLM\_c}])$$

$$+V\text{\_Cytosol} \cdot (\text{k\_tl\_GCLM\_mRNA\_c} \cdot [\text{GCLM\_mRNA\_c}])$$

$$\frac{\mathrm{d}([\text{GCLM\_mRNA\_c}] \cdot V\text{\_Cytosol})}{\mathrm{d}t} = +V\text{\_Cytosol} \cdot \left(\frac{\text{k\_ts\_GCLM\_gene,a\_n} \cdot [\text{GCLM\_gene,a\_n}] \cdot \frac{1}{3} \cdot V\text{\_Nucleus}}{V\text{\_Cytosol}}\right)$$

$$-V\text{\_Cytosol} \cdot (\text{k\_d\_GCLM\_mRNA\_c} \cdot [\text{GCLM\_mRNA\_c}])$$

$$\frac{\mathrm{d}([\text{GS,m\_c}] \cdot V\text{\_Cytosol})}{\mathrm{d}t} = -V\text{\_Cytosol} \cdot (\text{k\_d\_GS, m\_c} \cdot [\text{GS, m\_c}])$$

$$-2 \cdot V\text{\_Cytosol} \cdot ((\text{”k\_as\_GS, m\_c\_(GS, m\_c)”} \cdot [\text{GS, m\_c}] \cdot [\text{GS, m\_c}] - \text{k\_ds\_GS\_c} \cdot [\text{GS\_c}]))$$

$$+V\text{\_Cytosol} \cdot (\text{k\_tl\_GS\_mRNA\_c} \cdot [\text{GS\_mRNA\_c}])$$

$$\frac{\mathrm{d}([\text{GS\_c}] \cdot V\text{\_Cytosol})}{\mathrm{d}t} = -V\text{\_Cytosol} \cdot (\text{k\_d\_GS\_c} \cdot [\text{GS\_c}])$$

$$+V\text{\_Cytosol} \cdot ((\text{”k\_as\_GS, m\_c\_(GS, m\_c)”} \cdot [\text{GS, m\_c}] \cdot [\text{GS, m\_c}] - \text{k\_ds\_GS\_c} \cdot [\text{GS\_c}]))$$

$$\frac{\mathrm{d}([\text{GS\_mRNA\_c}] \cdot V\text{\_Cytosol})}{\mathrm{d}t} = +V\text{\_Cytosol} \cdot \left(\frac{\text{k\_ts\_GS\_gene,a\_n} \cdot [\text{GS\_gene,a\_n}] \cdot \frac{1}{3} \cdot V\text{\_Nucleus}}{V\text{\_Cytosol}}\right)$$

$$-V\text{\_Cytosol} \cdot (\text{k\_d\_GS\_mRNA\_c} \cdot [\text{GS\_mRNA\_c}])$$

$$
\begin{aligned}
\frac{\mathrm{d}([\text{GSH\_c}] \cdot V\text{\_Cytosol})}{\mathrm{d}t} = &- V\text{\_Cytosol} \cdot \left( \frac{\text{v\_cs\_GSH\_c} \cdot [\text{GSH\_c}]}{\text{Km\_cs\_GSH\_c} + [\text{GSH\_c}]} \right) \\
&+ V\text{\_Cytosol} \cdot \left( \text{k\_s\_GS\_c} \cdot [\text{GS\_c}] \cdot \frac{\frac{[\text{rGC\_c}]}{\text{Km\_s\_1\_rGC\_c\_@GS\_c}} + \frac{[\text{rGC\_c}]^2}{\text{Km\_s\_1\_rGC\_c\_@GS\_c} \cdot \text{Km\_s\_2\_rGC\_c\_@GS\_c}}}{1 + \frac{2 \cdot [\text{rGC\_c}]}{\text{Km\_s\_1\_rGC\_c\_@GS\_c}} + \frac{[\text{rGC\_c}]^2}{\text{Km\_s\_1\_rGC\_c\_@GS\_c} \cdot \text{Km\_s\_2\_rGC\_c\_@GS\_c}}} \cdot \frac{[\text{Gly\_c}]}{\text{Km\_s\_Gly\_c\_@GS\_c} + [\text{Gly\_c}]} \cdot \frac{[\text{ATP\_c}]}{\text{Km\_s\_ATP\_c\_@GS\_c} + [\text{ATP\_c}]} \right) \\
&- V\text{\_Cytosol} \cdot \left( \frac{\text{"k\_cj\_X}'\text{\_c\_(GSH\_c)\_@GST\_c"} \cdot [\text{X}'\text{\_c}]}{[\text{X}'\text{\_c}] + [\text{X}'\text{b\_c}]} \cdot [\text{GST\_c}] \cdot \frac{[\text{GSH\_c}]}{\text{Km\_cj\_GSH\_c\_@GST\_c} \cdot \left(1 + \frac{[\text{X}''\text{\_c}]}{\text{Ki\_in\_X}''\text{\_c\_@GST\_c}}\right) + [\text{GSH\_c}]} \right. \\
&\left. \cdot \frac{[\text{X}'\text{\_c}]}{(\text{Km\_cj\_X}'\text{\_c\_@GST\_c} + [\text{X}'\text{\_c}]) \cdot \left(1 + \frac{[\text{X}''\text{\_c}]}{\text{Ki\_in\_X}''\text{\_c\_@GST\_c}}\right)} \cdot 1 \right) \\
&- V\text{\_Cytosol} \cdot \left( \frac{\text{"k\_cj\_X}'\text{b\_c\_(GSH\_c)\_@GST\_c"} \cdot [\text{X}'\text{b\_c}]}{[\text{X}'\text{b\_c}] + [\text{X}'\text{\_c}]} \cdot [\text{GST\_c}] \cdot \frac{[\text{GSH\_c}]}{\text{Km\_cj\_6\_GSH\_c\_@GST\_c} \cdot \left(1 + \frac{[\text{X}''\text{b\_c}]}{\text{Ki\_in\_X}''\text{b\_c\_@GST\_c}}\right) + [\text{GSH\_c}]} \right. \\
&\left. \cdot \frac{[\text{X}'\text{b\_c}]}{(\text{Km\_cj\_X}'\text{b\_c\_@GST\_c} + [\text{X}'\text{b\_c}]) \cdot \left(1 + \frac{[\text{X}''\text{b\_c}]}{\text{Ki\_in\_X}''\text{b\_c\_@GST\_c}}\right)} \cdot 1 \right)
\end{aligned}
$$

$$
\begin{aligned}
\frac{\mathrm{d}([\text{GST,m\_c}] \cdot V\text{\_Cytosol})}{\mathrm{d}t} = &- V\text{\_Cytosol} \cdot (\text{k\_d\_GST, m\_c} \cdot [\text{GST, m\_c}]) \\
&- 2 \cdot V\text{\_Cytosol} \cdot ((\text{"k\_as\_GST, m\_c\_(GST, m\_c)"} \cdot [\text{GST, m\_c}] \cdot [\text{GST, m\_c}] - \text{k\_ds\_GST\_c} \cdot [\text{GST\_c}])) \\
&+ V\text{\_Cytosol} \cdot (\text{k\_tl\_GST\_mRNA\_c} \cdot [\text{GST\_mRNA\_c}])
\end{aligned}
$$

$$
\begin{aligned}
\frac{\mathrm{d}([\text{GST\_c}] \cdot V\text{\_Cytosol})}{\mathrm{d}t} = &- V\text{\_Cytosol} \cdot (\text{k\_d\_GST\_c} \cdot [\text{GST\_c}]) \\
&+ V\text{\_Cytosol} \cdot ((\text{"k\_as\_GST, m\_c\_(GST, m\_c)"} \cdot [\text{GST, m\_c}] \cdot [\text{GST, m\_c}] - \text{k\_ds\_GST\_c} \cdot [\text{GST\_c}]))
\end{aligned}
$$

$$
\begin{aligned}
\frac{\mathrm{d}([\text{GST\_mRNA\_c}] \cdot V\text{\_Cytosol})}{\mathrm{d}t} = &+ V\text{\_Cytosol} \cdot \left( \frac{\text{k\_ts\_GST\_gene,a\_n} \cdot [\text{GST\_gene,a\_n}] \cdot \frac{1}{3} \cdot V\text{\_Nucleus}}{V\text{\_Cytosol}} \right) \\
&- V\text{\_Cytosol} \cdot (\text{k\_d\_GST\_mRNA\_c} \cdot [\text{GST\_mRNA\_c}])
\end{aligned}
$$

$$
\begin{aligned}
\frac{\mathrm{d}([\text{Keap1,o\_c}] \cdot V\text{\_Cytosol})}{\mathrm{d}t} = &- V\text{\_Cytosol} \cdot (\text{k\_rd\_Keap1, o\_c} \cdot [\text{Keap1, o\_c}]) \\
&+ V\text{\_Cytosol} \cdot ([\text{Keap1\_c}] \cdot (\text{k\_ox\_b\_Keap1\_c} + ([\text{X}'\text{\_c}] + [\text{X}'\text{b\_c}]) \cdot \text{"k\_ox\_Keap1\_c\_(X}'\text{\_c)"})) \\
&+ V\text{\_Cytosol} \cdot (\text{k\_d\_Nrf2:Keap1, o\_c} \cdot [\text{Nrf2:Keap1, o\_c}]) \\
&- V\text{\_Cytosol} \cdot ((\text{"k\_as\_Nrf2\_c\_(Keap1, o\_c)"} \cdot [\text{Keap1, o\_c}] \cdot [\text{Nrf2\_c}] - \text{k\_ds\_Nrf2:Keap1, o\_c} \cdot [\text{Nrf2:Keap1, o\_c}]))
\end{aligned}
$$

$$\frac{\mathrm{d}([\text{Keap1\_c}] \cdot V\text{\_Cytosol})}{\mathrm{d}t} = +V\text{\_Cytosol} \cdot (\text{k\_rd\_Keap1, o\_c} \cdot [\text{Keap1, o\_c}])$$
$$-V\text{\_Cytosol} \cdot ([\text{Keap1\_c}] \cdot (\text{k\_ox\_b\_Keap1\_c} + ([\text{X}'\text{\_c}] + [\text{X}'\text{b\_c}]) \cdot \text{"k\_ox\_Keap1\_c\_(X}'\text{\_c)"}))$$
$$+V\text{\_Cytosol} \cdot (\text{k\_d\_Nrf2:Keap1\_c} \cdot [\text{Nrf2:Keap1\_c}])$$
$$-V\text{\_Cytosol} \cdot ((\text{"k\_as\_Nrf2\_c\_(Keap1\_c)"} \cdot [\text{Keap1\_c}] \cdot [\text{Nrf2\_c}] - \text{k\_ds\_Nrf2:Keap1\_c} \cdot [\text{Nrf2:Keap1\_c}]))$$

$$\frac{\mathrm{d}([\text{NR\_c}] \cdot V\text{\_Cytosol})}{\mathrm{d}t} = -V\text{\_Cytosol} \cdot ((\text{"k\_as\_X\_c\_(NR\_c)"} \cdot [\text{X\_c}] \cdot [\text{NR\_c}] - \text{k\_ds\_XNR\_c} \cdot [\text{XNR\_c}]))$$

$$\frac{\mathrm{d}([\text{Nrf2:Keap1,o\_c}] \cdot V\text{\_Cytosol})}{\mathrm{d}t} = -V\text{\_Cytosol} \cdot (\text{k\_d\_Nrf2:Keap1, o\_c} \cdot [\text{Nrf2:Keap1, o\_c}])$$
$$+V\text{\_Cytosol} \cdot ((\text{"k\_as\_Nrf2\_c\_(Keap1, o\_c)"} \cdot [\text{Keap1, o\_c}] \cdot [\text{Nrf2\_c}] - \text{k\_ds\_Nrf2:Keap1, o\_c} \cdot [\text{Nrf2:Keap1, o\_c}]))$$
$$-V\text{\_Cytosol} \cdot (\text{k\_rd\_Nrf2:Keap1, o\_c} \cdot [\text{Nrf2:Keap1, o\_c}])$$
$$+V\text{\_Cytosol} \cdot ([\text{Nrf2:Keap1\_c}] \cdot (\text{k\_ox\_b\_Nrf2:Keap1\_c} + ([\text{X}'\text{\_c}] + [\text{X}'\text{b\_c}]) \cdot \text{"k\_ox\_Nrf2:Keap1\_c\_(X}'\text{\_c)"}))$$

$$\frac{\mathrm{d}([\text{Nrf2:Keap1\_c}] \cdot V\text{\_Cytosol})}{\mathrm{d}t} = +V\text{\_Cytosol} \cdot (\text{k\_rd\_Nrf2:Keap1, o\_c} \cdot [\text{Nrf2:Keap1, o\_c}])$$
$$-V\text{\_Cytosol} \cdot (\text{k\_d\_Nrf2:Keap1\_c} \cdot [\text{Nrf2:Keap1\_c}])$$
$$+V\text{\_Cytosol} \cdot ((\text{"k\_as\_Nrf2\_c\_(Keap1\_c)"} \cdot [\text{Keap1\_c}] \cdot [\text{Nrf2\_c}] - \text{k\_ds\_Nrf2:Keap1\_c} \cdot [\text{Nrf2:Keap1\_c}]))$$
$$-V\text{\_Cytosol} \cdot ([\text{Nrf2:Keap1\_c}] \cdot (\text{k\_ox\_b\_Nrf2:Keap1\_c} + ([\text{X}'\text{\_c}] + [\text{X}'\text{b\_c}]) \cdot \text{"k\_ox\_Nrf2:Keap1\_c\_(X}'\text{\_c)"}))$$

$$\frac{\mathrm{d}([\text{Nrf2\_c}] \cdot V\text{\_Cytosol})}{\mathrm{d}t} = -V\text{\_Cytosol} \cdot ((\text{"k\_as\_Nrf2\_c\_(Keap1, o\_c)"} \cdot [\text{Keap1, o\_c}] \cdot [\text{Nrf2\_c}] - \text{k\_ds\_Nrf2:Keap1, o\_c} \cdot [\text{Nrf2:Keap1, o\_c}]))$$
$$-V\text{\_Cytosol} \cdot ((\text{"k\_as\_Nrf2\_c\_(Keap1\_c)"} \cdot [\text{Keap1\_c}] \cdot [\text{Nrf2\_c}] - \text{k\_ds\_Nrf2:Keap1\_c} \cdot [\text{Nrf2:Keap1\_c}]))$$
$$-V\text{\_Cytosol} \cdot (\text{k\_d\_Nrf2\_c} \cdot [\text{Nrf2\_c}])$$
$$-((\text{k\_tn\_Nrf2\_c} \cdot [\text{Nrf2\_c}] - \text{k\_tn\_Nrf2\_n} \cdot [\text{Nrf2\_n}]) \cdot V\text{\_Nucleus})$$
$$+V\text{\_Cytosol} \cdot (\text{k\_tl\_Nrf2\_mRNA\_c} \cdot [\text{Nrf2\_mRNA\_c}])$$

$$\frac{\mathrm{d}([\text{Nrf2\_mRNA\_c}] \cdot V\text{\_Cytosol})}{\mathrm{d}t} = +V\text{\_Cytosol} \cdot \left(\frac{\text{k\_ts\_Nrf2\_gene,a\_n} \cdot [\text{Nrf2\_gene,a\_n}] \cdot \frac{1}{3} \cdot V\text{\_Nucleus}}{V\text{\_Cytosol}}\right)$$
$$-V\text{\_Cytosol} \cdot (\text{k\_d\_Nrf2\_mRNA\_c} \cdot [\text{Nrf2\_mRNA\_c}] \cdot 1)$$

$$
\begin{aligned}
\frac{\mathrm{d}([\text{rGC\_c}]\cdot V\text{\_Cytosol})}{\mathrm{d}t} &= -V\text{\_Cytosol}\cdot\Bigg(\text{k\_s\_GS\_c}\cdot[\text{GS\_c}]\cdot\frac{\frac{[\text{rGC\_c}]}{\text{Km\_s\_1\_rGC\_c\_@GS\_c}}+\frac{[\text{rGC\_c}]^2}{\text{Km\_s\_1\_rGC\_c\_@GS\_c}\cdot\text{Km\_s\_2\_rGC\_c\_@GS\_c}}}{1+\frac{2\cdot[\text{rGC\_c}]}{\text{Km\_s\_1\_rGC\_c\_@GS\_c}}+\frac{[\text{rGC\_c}]^2}{\text{Km\_s\_1\_rGC\_c\_@GS\_c}\cdot\text{Km\_s\_2\_rGC\_c\_@GS\_c}}} \\
&\quad\cdot\frac{[\text{Gly\_c}]}{\text{Km\_s\_Gly\_c\_@GS\_c}+[\text{Gly\_c}]}\cdot\frac{[\text{ATP\_c}]}{\text{Km\_s\_ATP\_c\_@GS\_c}+[\text{ATP\_c}]}\Bigg) \\
&\quad+V\text{\_Cytosol}\cdot\Bigg(\text{k\_s\_GCL\_c}\cdot[\text{GCL\_c}]\cdot\frac{[\text{Cys\_c}]}{\text{Km\_s\_Cys\_c\_@GCL\_c}+[\text{Cys\_c}]} \\
&\quad\cdot\frac{[\text{Glu\_c}]}{\text{Km\_s\_Glu\_c\_@GCL\_c}\cdot\left(\text{P\_s\_rGC\_c}+\frac{[\text{GSH\_c}]}{\text{Ki\_in\_2}'\text{\_GSH\_c\_@GCL\_c}}\right)+[\text{Glu\_c}]\cdot\left(\text{P\_s\_rGC\_c}+\frac{[\text{GSH\_c}]}{\text{Ki\_in\_1}'\text{\_GSH\_c\_@GCL\_c}}\right)} \\
&\quad\cdot\frac{[\text{ATP\_c}]}{\text{Km\_s\_ATP\_c\_@GCL\_c}\cdot\left(\text{P\_s\_rGC\_c}+\frac{[\text{GSH\_c}]}{\text{Ki\_in\_2\_GSH\_c\_@GCL\_c}}\right)+[\text{ATP\_c}]\cdot\left(\text{P\_s\_rGC\_c}+\frac{[\text{GSH\_c}]}{\text{Ki\_in\_1\_GSH\_c\_@GCL\_c}}\right)} \\
&\quad+\text{k\_s\_GCLC\_c}\cdot[\text{GCLC\_c}]\cdot\frac{[\text{Cys\_c}]}{\text{Km\_s\_Cys\_c\_@GCLC\_c}+[\text{Cys\_c}]} \\
&\quad\cdot\frac{[\text{Glu\_c}]}{\text{Km\_s\_Glu\_c\_@GCLC\_c}\cdot\left(\text{P\_s\_rGC\_c}+\frac{[\text{GSH\_c}]}{\text{Ki\_in\_2}'\text{\_GSH\_c\_@GCLC\_c}}\right)+[\text{Glu\_c}]\cdot\left(\text{P\_s\_rGC\_c}+\frac{[\text{GSH\_c}]}{\text{Ki\_in\_1}'\text{\_GSH\_c\_@GCLC\_c}}\right)} \\
&\quad\cdot\frac{[\text{ATP\_c}]}{\text{Km\_s\_ATP\_c\_@GCLC\_c}\cdot\left(\text{P\_s\_rGC\_c}+\frac{[\text{GSH\_c}]}{\text{Ki\_in\_2\_GSH\_c\_@GCLC\_c}}\right)+[\text{ATP\_c}]\cdot\left(\text{P\_s\_rGC\_c}+\frac{[\text{GSH\_c}]}{\text{Ki\_in\_1\_GSH\_c\_@GCLC\_c}}\right)}\Bigg)
\end{aligned}
$$

$$
\begin{aligned}
\frac{\mathrm{d}\left([\text{X}''\text{\_c}]\cdot V\text{\_Cytosol}\right)}{\mathrm{d}t} &= -\left(\frac{\text{k\_tp\_X}''\text{\_c\_@ABC3\_c}\cdot[\text{ABC3\_c}]\cdot\frac{[\text{X}''\text{\_c}]}{[\text{X}''\text{\_c}]+[\text{X}''\text{b\_c}]}\cdot[\text{X}''\text{\_c}]}{\text{Km\_tp\_X}''\text{\_c\_@ABC3\_c}+[\text{X}''\text{\_c}]}\cdot V\text{\_Cytosol}\right) \\
&\quad+V\text{\_Cytosol}\cdot\Bigg(\frac{\text{"k\_cj\_X}'\text{\_c\_(GSH\_c)\_@GST\_c"}\cdot[\text{X}'\text{\_c}]}{[\text{X}'\text{\_c}]+[\text{X}'\text{b\_c}]}\cdot[\text{GST\_c}]\cdot\frac{[\text{GSH\_c}]}{\text{Km\_cj\_GSH\_c\_@GST\_c}\cdot\left(1+\frac{[\text{X}''\text{\_c}]}{\text{Ki\_in\_X}''\text{\_c\_@GST\_c}}\right)+[\text{GSH\_c}]} \\
&\quad\cdot\frac{[\text{X}'\text{\_c}]}{(\text{Km\_cj\_X}'\text{\_c\_@GST\_c}+[\text{X}'\text{\_c}])\cdot\left(1+\frac{[\text{X}''\text{\_c}]}{\text{Ki\_in\_X}''\text{\_c\_@GST\_c}}\right)}\cdot 1\Bigg)
\end{aligned}
$$

$$
\begin{aligned}
\frac{\mathrm{d}\left([\text{X}''\text{b\_c}]\cdot V\text{\_Cytosol}\right)}{\mathrm{d}t} &= -\left(\frac{\text{k\_tp\_X}''\text{b\_c\_@ABC3\_c}\cdot[\text{ABC3\_c}]\cdot\frac{[\text{X}''\text{b\_c}]}{[\text{X}''\text{b\_c}]+[\text{X}''\text{\_c}]}\cdot[\text{X}''\text{b\_c}]}{\text{Km\_tp\_X}''\text{b\_c\_@ABC3\_c}+[\text{X}''\text{b\_c}]}\cdot V\text{\_Cytosol}\right) \\
&\quad+V\text{\_Cytosol}\cdot\Bigg(\frac{\text{"k\_cj\_X}'\text{b\_c\_(GSH\_c)\_@GST\_c"}\cdot[\text{X}'\text{b\_c}]}{[\text{X}'\text{b\_c}]+[\text{X}'\text{\_c}]}\cdot[\text{GST\_c}]\cdot\frac{[\text{GSH\_c}]}{\text{Km\_cj\_6\_GSH\_c\_@GST\_c}\cdot\left(1+\frac{[\text{X}''\text{b\_c}]}{\text{Ki\_in\_X}''\text{b\_c\_@GST\_c}}\right)+[\text{GSH\_c}]} \\
&\quad\cdot\frac{[\text{X}'\text{b\_c}]}{(\text{Km\_cj\_X}'\text{b\_c\_@GST\_c}+[\text{X}'\text{b\_c}])\cdot\left(1+\frac{[\text{X}''\text{b\_c}]}{\text{Ki\_in\_X}''\text{b\_c\_@GST\_c}}\right)}\cdot 1\Bigg)
\end{aligned}
$$

$$\frac{d\left([X'\_c]\cdot V\_Cytosol\right)}{dt} = -V\_Cytosol\cdot\left(\frac{\text{"k\_cj\_X'\_c\_(GSH\_c)\_@GST\_c"}\cdot[X'\_c]}{[X'\_c]+[X'b\_c]}\cdot[GST\_c]\cdot\frac{[GSH\_c]}{Km\_cj\_GSH\_c\_@GST\_c\cdot\left(1+\frac{[X''\_c]}{Ki\_in\_X''\_c\_@GST\_c}\right)+[GSH\_c]}\right.$$

$$\left.\cdot\frac{[X'\_c]}{(Km\_cj\_X'\_c\_@GST\_c+[X'\_c])\cdot\left(1+\frac{[X''\_c]}{Ki\_in\_X''\_c\_@GST\_c}\right)}\cdot 1\right)$$

$$+V\_Cytosol\cdot\left(\frac{k\_ox\_X\_c\_@CYP\_c\cdot[CYP\_c]\cdot[X\_c]}{Km\_ox\_X\_c\_@CYP\_c+[X\_c]}\right)$$

$$\frac{d\left([X'b\_c]\cdot V\_Cytosol\right)}{dt} = -V\_Cytosol\cdot\left(\frac{\text{"k\_cj\_X'b\_c\_(GSH\_c)\_@GST\_c"}\cdot[X'b\_c]}{[X'b\_c]+[X'\_c]}\cdot[GST\_c]\cdot\frac{[GSH\_c]}{Km\_cj\_6\_GSH\_c\_@GST\_c\cdot\left(1+\frac{[X''b\_c]}{Ki\_in\_X''b\_c\_@GST\_c}\right)+[GSH\_c]}\right.$$

$$\left.\cdot\frac{[X'b\_c]}{(Km\_cj\_X'b\_c\_@GST\_c+[X'b\_c])\cdot\left(1+\frac{[X''b\_c]}{Ki\_in\_X''b\_c\_@GST\_c}\right)}\cdot 1\right)$$

$$+V\_Cytosol\cdot(k\_pr\_b)$$

$$\frac{d([X\_c]\cdot V\_Cytosol)}{dt} = -\left((k\_df\_1\_X\_c\cdot[X\_c]-k\_df\_X\_n\cdot[X\_n])\cdot V\_Nucleus\right)$$

$$-V\_Cytosol\cdot\left(\frac{k\_ox\_X\_c\_@CYP\_c\cdot[CYP\_c]\cdot[X\_c]}{Km\_ox\_X\_c\_@CYP\_c+[X\_c]}\right)$$

$$-\left(\frac{k\_tp\_X\_c\_@ABC0\_c\cdot[ABC0\_c]\cdot[X\_c]}{Km\_tp\_X\_c\_@ABC0\_c+[X\_c]}\cdot V\_Cytosol\right)$$

$$+\left((k\_df\_X\_e\cdot[X\_e]-k\_df\_2\_X\_c\cdot[X\_c])\cdot V\_Cytosol\right)$$

$$-V\_Cytosol\cdot\left((\text{"k\_as\_X\_c\_(NR\_c)"}\cdot[X\_c]\cdot[NR\_c]-k\_ds\_XNR\_c\cdot[XNR\_c])\right)$$

$$\frac{d([XNR\_c]\cdot V\_Cytosol)}{dt} = +V\_Cytosol\cdot\left((\text{"k\_as\_X\_c\_(NR\_c)"}\cdot[X\_c]\cdot[NR\_c]-k\_ds\_XNR\_c\cdot[XNR\_c])\right)$$

$$-\left((k\_tn\_XNR\_c\cdot[XNR\_c]-k\_tn\_XNR\_n\cdot[XNR\_n])\cdot V\_Nucleus\right)$$

$$\frac{d\left([X''\_e]\cdot V\_Extracellular\right)}{dt} = +\left(\frac{k\_tp\_X''\_c\_@ABC3\_c\cdot[ABC3\_c]\cdot\frac{[X''\_c]}{[X''\_c]+[X''b\_c]}\cdot[X''\_c]}{Km\_tp\_X''\_c\_@ABC3\_c+[X''\_c]}\cdot V\_Cytosol\right)$$

$$\frac{d\left([X''b\_e]\cdot V\_Extracellular\right)}{dt} = +\left(\frac{k\_tp\_X''b\_c\_@ABC3\_c\cdot[ABC3\_c]\cdot\frac{[X''b\_c]}{[X''b\_c]+[X''\_c]}\cdot[X''b\_c]}{Km\_tp\_X''b\_c\_@ABC3\_c+[X''b\_c]}\cdot V\_Cytosol\right)$$

$$\frac{d([ABC0\_ARE\_n]\cdot V\_Nucleus)}{dt} = -V\_Nucleus\cdot\left(\text{"k\_as\_Nrf2:Maf\_n\_(ABC0\_ARE\_n)"}\cdot[ABC0\_ARE\_n]\cdot[Nrf2{:}Maf\_n]^{\text{"H\_as\_Nrf2:Maf\_n\_(ABC0\_ARE\_n)"}}\right.$$

$$\left.-k\_ds\_ABC0\_NMA\_n\cdot[ABC0\_NMA\_n]\right)$$

$$\frac{\mathrm{d}([\mathrm{ABC0\_DRE\_n}] \cdot V\mathrm{\_Nucleus})}{\mathrm{d}t} = -V\mathrm{\_Nucleus} \cdot ((\mathrm{"k\_as\_XNRT\_n\_(ABC0\_DRE\_n)"} \cdot [\mathrm{XNRT\_n}] \cdot [\mathrm{ABC0\_DRE\_n}] - \mathrm{k\_ds\_ABC0\_XNRTD\_n} \cdot [\mathrm{ABC0\_XNRTD\_n}]))$$

$$\frac{\mathrm{d}([\mathrm{ABC0\_gene,a\_n}] \cdot V\mathrm{\_Nucleus})}{\mathrm{d}t} = +V\mathrm{\_Nucleus} \cdot \big(\mathrm{k\_a\_b\_ABC0\_gene,i\_n} \cdot [\mathrm{ABC0\_gene,i\_n}] + \mathrm{"k\_a\_ABC0\_gene,i\_n\_(ABC0\_NMA\_n)"} \cdot [\mathrm{ABC0\_gene,i\_n}] \cdot [\mathrm{ABC0\_NMA\_n}] + \mathrm{"k\_a\_ABC0\_gene,i\_n\_(ABC0\_XNRTD\_n)"} \cdot [\mathrm{ABC0\_gene,i\_n}] \cdot [\mathrm{ABC0\_XNRTD\_n}] - \mathrm{k\_i\_ABC0\_gene,a\_n} \cdot [\mathrm{ABC0\_gene,a\_n}]\big)$$

$$\frac{\mathrm{d}([\mathrm{ABC0\_gene,i\_n}] \cdot V\mathrm{\_Nucleus})}{\mathrm{d}t} = -V\mathrm{\_Nucleus} \cdot \big(\mathrm{k\_a\_b\_ABC0\_gene,i\_n} \cdot [\mathrm{ABC0\_gene,i\_n}] + \mathrm{"k\_a\_ABC0\_gene,i\_n\_(ABC0\_NMA\_n)"} \cdot [\mathrm{ABC0\_gene,i\_n}] \cdot [\mathrm{ABC0\_NMA\_n}] + \mathrm{"k\_a\_ABC0\_gene,i\_n\_(ABC0\_XNRTD\_n)"} \cdot [\mathrm{ABC0\_gene,i\_n}] \cdot [\mathrm{ABC0\_XNRTD\_n}] - \mathrm{k\_i\_ABC0\_gene,a\_n} \cdot [\mathrm{ABC0\_gene,a\_n}]\big)$$

$$\frac{\mathrm{d}([\mathrm{ABC0\_NMA\_n}] \cdot V\mathrm{\_Nucleus})}{\mathrm{d}t} = +V\mathrm{\_Nucleus} \cdot \Big(\mathrm{"k\_as\_Nrf2{:}Maf\_n\_(ABC0\_ARE\_n)"} \cdot [\mathrm{ABC0\_ARE\_n}] \cdot [\mathrm{Nrf2{:}Maf\_n}]^{\mathrm{"H\_as\_Nrf2{:}Maf\_n\_(ABC0\_ARE\_n)"}} - \mathrm{k\_ds\_ABC0\_NMA\_n} \cdot [\mathrm{ABC0\_NMA\_n}]\Big)$$

$$\frac{\mathrm{d}([\mathrm{ABC0\_XNRTD\_n}] \cdot V\mathrm{\_Nucleus})}{\mathrm{d}t} = +V\mathrm{\_Nucleus} \cdot ((\mathrm{"k\_as\_XNRT\_n\_(ABC0\_DRE\_n)"} \cdot [\mathrm{XNRT\_n}] \cdot [\mathrm{ABC0\_DRE\_n}] - \mathrm{k\_ds\_ABC0\_XNRTD\_n} \cdot [\mathrm{ABC0\_XNRTD\_n}]))$$

$$\frac{\mathrm{d}([\mathrm{ABC3\_ARE\_n}] \cdot V\mathrm{\_Nucleus})}{\mathrm{d}t} = -V\mathrm{\_Nucleus} \cdot \Big(\mathrm{"k\_as\_Nrf2{:}Maf\_n\_(ABC3\_ARE\_n)"} \cdot [\mathrm{ABC3\_ARE\_n}] \cdot [\mathrm{Nrf2{:}Maf\_n}]^{\mathrm{"H\_as\_Nrf2{:}Maf\_n\_(ABC3\_ARE\_n)"}} - \mathrm{k\_ds\_ABC3\_NMA\_n} \cdot [\mathrm{ABC3\_NMA\_n}]\Big)$$

$$\frac{\mathrm{d}([\mathrm{ABC3\_DRE\_n}] \cdot V\mathrm{\_Nucleus})}{\mathrm{d}t} = -V\mathrm{\_Nucleus} \cdot ((\mathrm{"k\_as\_XNRT\_n\_(ABC3\_DRE\_n)"} \cdot [\mathrm{XNRT\_n}] \cdot [\mathrm{ABC3\_DRE\_n}] - \mathrm{k\_ds\_ABC3\_XNRTD\_n} \cdot [\mathrm{ABC3\_XNRTD\_n}]))$$

$$\frac{\mathrm{d}([\mathrm{ABC3\_gene,a\_n}] \cdot V\mathrm{\_Nucleus})}{\mathrm{d}t} = +V\mathrm{\_Nucleus} \cdot \big(\mathrm{k\_a\_b\_ABC3\_gene,i\_n} \cdot [\mathrm{ABC3\_gene,i\_n}] + \mathrm{"k\_a\_ABC3\_gene,i\_n\_(ABC3\_NMA\_n)"} \cdot [\mathrm{ABC3\_gene,i\_n}] \cdot [\mathrm{ABC3\_NMA\_n}] + \mathrm{"k\_a\_ABC3\_gene,i\_n\_(ABC3\_XNRTD\_n)"} \cdot [\mathrm{ABC3\_gene,i\_n}] \cdot [\mathrm{ABC3\_XNRTD\_n}] - \mathrm{k\_i\_ABC3\_gene,a\_n} \cdot [\mathrm{ABC3\_gene,a\_n}]\big)$$

$$\frac{\mathrm{d}([\mathrm{ABC3\_gene,i\_n}] \cdot V\mathrm{\_Nucleus})}{\mathrm{d}t} = -V\mathrm{\_Nucleus} \cdot \big(\mathrm{k\_a\_b\_ABC3\_gene,i\_n} \cdot [\mathrm{ABC3\_gene,i\_n}] + \mathrm{"k\_a\_ABC3\_gene,i\_n\_(ABC3\_NMA\_n)"} \cdot [\mathrm{ABC3\_gene,i\_n}] \cdot [\mathrm{ABC3\_NMA\_n}] + \mathrm{"k\_a\_ABC3\_gene,i\_n\_(ABC3\_XNRTD\_n)"} \cdot [\mathrm{ABC3\_gene,i\_n}] \cdot [\mathrm{ABC3\_XNRTD\_n}] - \mathrm{k\_i\_ABC3\_gene,a\_n} \cdot [\mathrm{ABC3\_gene,a\_n}]\big)$$

$$\frac{\mathrm{d}([\mathrm{ABC3\_NMA\_n}] \cdot V\mathrm{\_Nucleus})}{\mathrm{d}t} = +V\mathrm{\_Nucleus} \cdot \Big(\mathrm{"k\_as\_Nrf2{:}Maf\_n\_(ABC3\_ARE\_n)"} \cdot [\mathrm{ABC3\_ARE\_n}] \cdot [\mathrm{Nrf2{:}Maf\_n}]^{\mathrm{"H\_as\_Nrf2{:}Maf\_n\_(ABC3\_ARE\_n)"}} - \mathrm{k\_ds\_ABC3\_NMA\_n} \cdot [\mathrm{ABC3\_NMA\_n}]\Big)$$

$$\frac{\mathrm{d}([\mathrm{ABC3\_XNRTD\_n}] \cdot V\mathrm{\_Nucleus})}{\mathrm{d}t} = +V\mathrm{\_Nucleus} \cdot ((\mathrm{"k\_as\_XNRT\_n\_(ABC3\_DRE\_n)"} \cdot [\mathrm{XNRT\_n}] \cdot [\mathrm{ABC3\_DRE\_n}] - \mathrm{k\_ds\_ABC3\_XNRTD\_n} \cdot [\mathrm{ABC3\_XNRTD\_n}]))$$

$$\frac{\mathrm{d}([\mathrm{CYP\_DRE\_n}] \cdot V\mathrm{\_Nucleus})}{\mathrm{d}t} = -V\mathrm{\_Nucleus} \cdot \Big(\mathrm{"k\_as\_XNRT\_n\_(CYP\_DRE\_n)"} \cdot [\mathrm{CYP\_DRE\_n}] \cdot [\mathrm{XNRT\_n}]^{\mathrm{"H\_as\_XNRT\_n\_(CYP\_DRE\_n)"}} - \mathrm{k\_ds\_CYP\_XNRTD\_n} \cdot [\mathrm{CYP\_XNRTD\_n}]\Big)$$

$$\frac{d([CYP\_gene,a\_n] \cdot V\_Nucleus)}{dt} = +V\_Nucleus \cdot \Big(k\_a\_b\_CYP\_gene,i\_n \cdot [CYP\_gene,i\_n] + "k\_a\_CYP\_gene,i\_n\_(CYP\_XNRTD\_n)" \cdot [CYP\_gene,i\_n] \cdot [CYP\_XNRTD\_n] - k\_i\_CYP\_gene,a\_n \cdot [CYP\_gene,a\_n]\Big)$$

$$\frac{d([CYP\_gene,i\_n] \cdot V\_Nucleus)}{dt} = -V\_Nucleus \cdot \Big(k\_a\_b\_CYP\_gene,i\_n \cdot [CYP\_gene,i\_n] + "k\_a\_CYP\_gene,i\_n\_(CYP\_XNRTD\_n)" \cdot [CYP\_gene,i\_n] \cdot [CYP\_XNRTD\_n] - k\_i\_CYP\_gene,a\_n \cdot [CYP\_gene,a\_n]\Big)$$

$$\frac{d([CYP\_XNRTD\_n] \cdot V\_Nucleus)}{dt} = +V\_Nucleus \cdot \Big("k\_as\_XNRT\_n\_(CYP\_DRE\_n)" \cdot [CYP\_DRE\_n] \cdot [XNRT\_n]^{"H\_as\_XNRT\_n\_(CYP\_DRE\_n)"} - k\_ds\_CYP\_XNRTD\_n \cdot [CYP\_XNRTD\_n]\Big)$$

$$\frac{d([GCLC\_ARE\_n] \cdot V\_Nucleus)}{dt} = -V\_Nucleus \cdot \Big("k\_as\_Nrf2{:}Maf\_n\_(GCLC\_ARE\_n)" \cdot [GCLC\_ARE\_n] \cdot [Nrf2{:}Maf\_n]^{"H\_as\_Nrf2{:}Maf\_n\_(GCLC\_ARE\_n)"} - k\_ds\_GCLC\_NMA\_n \cdot [GCLC\_NMA\_n]\Big)$$

$$\frac{d([GCLC\_gene,a\_n] \cdot V\_Nucleus)}{dt} = +V\_Nucleus \cdot \Big(k\_a\_b\_GCLC\_gene,i\_n \cdot [GCLC\_gene,i\_n] + "k\_a\_GCLC\_gene,i\_n\_(GCLC\_NMA\_n)" \cdot [GCLC\_gene,i\_n] \cdot [GCLC\_NMA\_n] - k\_i\_GCLC\_gene,a\_n \cdot [GCLC\_gene,a\_n]\Big)$$

$$\frac{d([GCLC\_gene,i\_n] \cdot V\_Nucleus)}{dt} = -V\_Nucleus \cdot \Big(k\_a\_b\_GCLC\_gene,i\_n \cdot [GCLC\_gene,i\_n] + "k\_a\_GCLC\_gene,i\_n\_(GCLC\_NMA\_n)" \cdot [GCLC\_gene,i\_n] \cdot [GCLC\_NMA\_n] - k\_i\_GCLC\_gene,a\_n \cdot [GCLC\_gene,a\_n]\Big)$$

$$\frac{d([GCLC\_NMA\_n] \cdot V\_Nucleus)}{dt} = +V\_Nucleus \cdot \Big("k\_as\_Nrf2{:}Maf\_n\_(GCLC\_ARE\_n)" \cdot [GCLC\_ARE\_n] \cdot [Nrf2{:}Maf\_n]^{"H\_as\_Nrf2{:}Maf\_n\_(GCLC\_ARE\_n)"} - k\_ds\_GCLC\_NMA\_n \cdot [GCLC\_NMA\_n]\Big)$$

$$\frac{d([GCLM\_ARE\_n] \cdot V\_Nucleus)}{dt} = -V\_Nucleus \cdot \Big("k\_as\_Nrf2{:}Maf\_n\_(GCLM\_ARE\_n)" \cdot [GCLM\_ARE\_n] \cdot [Nrf2{:}Maf\_n]^{"H\_as\_Nrf2{:}Maf\_n\_(GCLM\_ARE\_n)"} - k\_ds\_GCLM\_NMA\_n \cdot [GCLM\_NMA\_n]\Big)$$

$$\frac{d([GCLM\_gene,a\_n] \cdot V\_Nucleus)}{dt} = +V\_Nucleus \cdot \Big(k\_a\_b\_GCLM\_gene,i\_n \cdot [GCLM\_gene,i\_n] + "k\_a\_GCLM\_gene,i\_n\_(GCLM\_NMA\_n)" \cdot [GCLM\_gene,i\_n] \cdot [GCLM\_NMA\_n] - k\_i\_GCLM\_gene,a\_n \cdot [GCLM\_gene,a\_n]\Big)$$

$$\frac{d([GCLM\_gene,i\_n] \cdot V\_Nucleus)}{dt} = -V\_Nucleus \cdot \Big(k\_a\_b\_GCLM\_gene,i\_n \cdot [GCLM\_gene,i\_n] + "k\_a\_GCLM\_gene,i\_n\_(GCLM\_NMA\_n)" \cdot [GCLM\_gene,i\_n] \cdot [GCLM\_NMA\_n] - k\_i\_GCLM\_gene,a\_n \cdot [GCLM\_gene,a\_n]\Big)$$

$$\frac{\mathrm{d}([\text{GCLM\_NMA\_n}] \cdot V\text{\_Nucleus})}{\mathrm{d}t} = +V\text{\_Nucleus} \cdot \Big(\text{"k\_as\_Nrf2:Maf\_n\_(GCLM\_ARE\_n)"} \cdot [\text{GCLM\_ARE\_n}] \cdot [\text{Nrf2:Maf\_n}]^{\text{"H\_as\_Nrf2:Maf\_n\_(GCLM\_ARE\_n)"}} - \text{k\_ds\_GCLM\_NMA\_n} \cdot [\text{GCLM\_NMA\_n}]\Big)$$

$$\frac{\mathrm{d}([\text{GS\_ARE\_n}] \cdot V\text{\_Nucleus})}{\mathrm{d}t} = -V\text{\_Nucleus} \cdot \Big(\text{"k\_as\_Nrf2:Maf\_n\_(GS\_ARE\_n)"} \cdot [\text{GS\_ARE\_n}] \cdot [\text{Nrf2:Maf\_n}]^{\text{"H\_as\_Nrf2:Maf\_n\_(GS\_ARE\_n)"}} - \text{k\_ds\_GS\_NMA\_n} \cdot [\text{GS\_NMA\_n}]\Big)$$

$$\frac{\mathrm{d}([\text{GS\_gene,a\_n}] \cdot V\text{\_Nucleus})}{\mathrm{d}t} = +V\text{\_Nucleus} \cdot \big(\text{k\_a\_b\_GS\_gene, i\_n} \cdot [\text{GS\_gene, i\_n}] + \text{"k\_a\_GS\_gene, i\_n\_(GS\_NMA\_n)"} \cdot [\text{GS\_gene, i\_n}] \cdot [\text{GS\_NMA\_n}] - \text{k\_i\_GS\_gene, a\_n} \cdot [\text{GS\_gene, a\_n}]\big)$$

$$\frac{\mathrm{d}([\text{GS\_gene,i\_n}] \cdot V\text{\_Nucleus})}{\mathrm{d}t} = -V\text{\_Nucleus} \cdot \big(\text{k\_a\_b\_GS\_gene, i\_n} \cdot [\text{GS\_gene, i\_n}] + \text{"k\_a\_GS\_gene, i\_n\_(GS\_NMA\_n)"} \cdot [\text{GS\_gene, i\_n}] \cdot [\text{GS\_NMA\_n}] - \text{k\_i\_GS\_gene, a\_n} \cdot [\text{GS\_gene, a\_n}]\big)$$

$$\frac{\mathrm{d}([\text{GS\_NMA\_n}] \cdot V\text{\_Nucleus})}{\mathrm{d}t} = +V\text{\_Nucleus} \cdot \Big(\text{"k\_as\_Nrf2:Maf\_n\_(GS\_ARE\_n)"} \cdot [\text{GS\_ARE\_n}] \cdot [\text{Nrf2:Maf\_n}]^{\text{"H\_as\_Nrf2:Maf\_n\_(GS\_ARE\_n)"}} - \text{k\_ds\_GS\_NMA\_n} \cdot [\text{GS\_NMA\_n}]\Big)$$

$$\frac{\mathrm{d}([\text{GST\_ARE\_n}] \cdot V\text{\_Nucleus})}{\mathrm{d}t} = -V\text{\_Nucleus} \cdot \Big(\text{"k\_as\_Nrf2:Maf\_n\_(GST\_ARE\_n)"} \cdot [\text{GST\_ARE\_n}] \cdot [\text{Nrf2:Maf\_n}]^{\text{"H\_as\_Nrf2:Maf\_n\_(GST\_ARE\_n)"}} - \text{k\_ds\_GST\_NMA\_n} \cdot [\text{GST\_NMA\_n}]\Big)$$

$$\frac{\mathrm{d}([\text{GST\_DRE\_n}] \cdot V\text{\_Nucleus})}{\mathrm{d}t} = -V\text{\_Nucleus} \cdot \Big(\text{"k\_as\_XNRT\_n\_(GST\_DRE\_n)"} \cdot [\text{GST\_DRE\_n}] \cdot [\text{XNRT\_n}]^{\text{"H\_as\_XNRT\_n\_(GST\_DRE\_n)"}} - \text{k\_ds\_GST\_XNRTD\_n} \cdot [\text{GST\_XNRTD\_n}]\Big)$$

$$\frac{\mathrm{d}([\text{GST\_gene,a\_n}] \cdot V\text{\_Nucleus})}{\mathrm{d}t} = +V\text{\_Nucleus} \cdot \big(\text{k\_a\_b\_GST\_gene, i\_n} \cdot [\text{GST\_gene, i\_n}] + \text{"k\_a\_GST\_gene, i\_n\_(GST\_NMA\_n)"} \cdot [\text{GST\_gene, i\_n}] \cdot [\text{GST\_NMA\_n}] + \text{"k\_a\_GST\_gene, i\_n\_(GST\_XNRTD\_n)"} \cdot [\text{GST\_gene, i\_n}] \cdot [\text{GST\_XNRTD\_n}] - \text{k\_i\_GST\_gene, a\_n} \cdot [\text{GST\_gene, a\_n}]\big)$$

$$\frac{\mathrm{d}([\text{GST\_gene,i\_n}] \cdot V\text{\_Nucleus})}{\mathrm{d}t} = -V\text{\_Nucleus} \cdot \big(\text{k\_a\_b\_GST\_gene, i\_n} \cdot [\text{GST\_gene, i\_n}] + \text{"k\_a\_GST\_gene, i\_n\_(GST\_NMA\_n)"} \cdot [\text{GST\_gene, i\_n}] \cdot [\text{GST\_NMA\_n}] + \text{"k\_a\_GST\_gene, i\_n\_(GST\_XNRTD\_n)"} \cdot [\text{GST\_gene, i\_n}] \cdot [\text{GST\_XNRTD\_n}] - \text{k\_i\_GST\_gene, a\_n} \cdot [\text{GST\_gene, a\_n}]\big)$$

$$\frac{\mathrm{d}([\text{GST\_NMA\_n}] \cdot V\text{\_Nucleus})}{\mathrm{d}t} = +V\text{\_Nucleus} \cdot \Big(\text{"k\_as\_Nrf2:Maf\_n\_(GST\_ARE\_n)"} \cdot [\text{GST\_ARE\_n}] \cdot [\text{Nrf2:Maf\_n}]^{\text{"H\_as\_Nrf2:Maf\_n\_(GST\_ARE\_n)"}} - \text{k\_ds\_GST\_NMA\_n} \cdot [\text{GST\_NMA\_n}]\Big)$$

$$
\begin{aligned}
\frac{\mathrm{d}([\mathrm{GST\_XNRTD\_n}] \cdot V\mathrm{\_Nucleus})}{\mathrm{d}t} &= +V\mathrm{\_Nucleus} \cdot \Big(\text{”k\_as\_XNRT\_n\_(GST\_DRE\_n)”} \cdot [\mathrm{GST\_DRE\_n}] \cdot [\mathrm{XNRT\_n}]^{\text{”H\_as\_XNRT\_n\_(GST\_DRE\_n)”}} \\
&\quad - \mathrm{k\_ds\_GST\_XNRTD\_n} \cdot [\mathrm{GST\_XNRTD\_n}]\Big) \\
\frac{\mathrm{d}([\mathrm{Maf\_n}] \cdot V\mathrm{\_Nucleus})}{\mathrm{d}t} &= -V\mathrm{\_Nucleus} \cdot ((\text{”k\_as\_Nrf2\_n\_(Maf\_n)”} \cdot [\mathrm{Maf\_n}] \cdot [\mathrm{Nrf2\_n}] - \mathrm{k\_ds\_Nrf2{:}Maf\_n} \cdot [\mathrm{Nrf2{:}Maf\_n}])) \\
\frac{\mathrm{d}([\mathrm{NR\_n}] \cdot V\mathrm{\_Nucleus})}{\mathrm{d}t} &= -V\mathrm{\_Nucleus} \cdot ((\text{”k\_as\_X\_n\_(NR\_n)”} \cdot [\mathrm{NR\_n}] \cdot [\mathrm{X\_n}] - \mathrm{k\_ds\_XNR\_n} \cdot [\mathrm{XNR\_n}])) \\
\frac{\mathrm{d}([\mathrm{Nrf2{:}Maf\_n}] \cdot V\mathrm{\_Nucleus})}{\mathrm{d}t} &= -2 \cdot V\mathrm{\_Nucleus} \cdot \Big(\text{”k\_as\_Nrf2:Maf\_n\_(ABC3\_ARE\_n)”} \cdot [\mathrm{ABC3\_ARE\_n}] \cdot [\mathrm{Nrf2{:}Maf\_n}]^{\text{”H\_as\_Nrf2:Maf\_n\_(ABC3\_ARE\_n)”}} \\
&\quad - \mathrm{k\_ds\_ABC3\_NMA\_n} \cdot [\mathrm{ABC3\_NMA\_n}]\Big) \\
&\quad -3 \cdot V\mathrm{\_Nucleus} \cdot \Big(\text{”k\_as\_Nrf2:Maf\_n\_(GCLC\_ARE\_n)”} \cdot [\mathrm{GCLC\_ARE\_n}] \cdot [\mathrm{Nrf2{:}Maf\_n}]^{\text{”H\_as\_Nrf2:Maf\_n\_(GCLC\_ARE\_n)”}} \\
&\quad - \mathrm{k\_ds\_GCLC\_NMA\_n} \cdot [\mathrm{GCLC\_NMA\_n}]\Big) \\
&\quad -3 \cdot V\mathrm{\_Nucleus} \cdot \Big(\text{”k\_as\_Nrf2:Maf\_n\_(GCLM\_ARE\_n)”} \cdot [\mathrm{GCLM\_ARE\_n}] \cdot [\mathrm{Nrf2{:}Maf\_n}]^{\text{”H\_as\_Nrf2:Maf\_n\_(GCLM\_ARE\_n)”}} \\
&\quad - \mathrm{k\_ds\_GCLM\_NMA\_n} \cdot [\mathrm{GCLM\_NMA\_n}]\Big) \\
&\quad -2 \cdot V\mathrm{\_Nucleus} \cdot \Big(\text{”k\_as\_Nrf2:Maf\_n\_(GS\_ARE\_n)”} \cdot [\mathrm{GS\_ARE\_n}] \cdot [\mathrm{Nrf2{:}Maf\_n}]^{\text{”H\_as\_Nrf2:Maf\_n\_(GS\_ARE\_n)”}} - \mathrm{k\_ds\_GS\_NMA\_n} \cdot [\mathrm{GS\_NMA\_n}]\Big) \\
&\quad -2 \cdot V\mathrm{\_Nucleus} \cdot \Big(\text{”k\_as\_Nrf2:Maf\_n\_(GST\_ARE\_n)”} \cdot [\mathrm{GST\_ARE\_n}] \cdot [\mathrm{Nrf2{:}Maf\_n}]^{\text{”H\_as\_Nrf2:Maf\_n\_(GST\_ARE\_n)”}} - \mathrm{k\_ds\_GST\_NMA\_n} \cdot [\mathrm{GST\_NMA\_n}]\Big) \\
&\quad +V\mathrm{\_Nucleus} \cdot ((\text{”k\_as\_Nrf2\_n\_(Maf\_n)”} \cdot [\mathrm{Maf\_n}] \cdot [\mathrm{Nrf2\_n}] - \mathrm{k\_ds\_Nrf2{:}Maf\_n} \cdot [\mathrm{Nrf2{:}Maf\_n}])) \\
&\quad -V\mathrm{\_Nucleus} \cdot \Big(\text{”k\_as\_Nrf2:Maf\_n\_(ABC0\_ARE\_n)”} \cdot [\mathrm{ABC0\_ARE\_n}] \cdot [\mathrm{Nrf2{:}Maf\_n}]^{\text{”H\_as\_Nrf2:Maf\_n\_(ABC0\_ARE\_n)”}} \\
&\quad - \mathrm{k\_ds\_ABC0\_NMA\_n} \cdot [\mathrm{ABC0\_NMA\_n}]\Big) \\
&\quad -V\mathrm{\_Nucleus} \cdot ((\text{”k\_as\_Nrf2:Maf\_n\_(Nrf2\_ARE\_n)”} \cdot [\mathrm{Nrf2{:}Maf\_n}] \cdot [\mathrm{Nrf2\_ARE\_n}] - \mathrm{k\_ds\_Nrf2\_NMA\_n} \cdot [\mathrm{Nrf2\_NMA\_n}])) \\
\frac{\mathrm{d}([\mathrm{Nrf2\_ARE\_n}] \cdot V\mathrm{\_Nucleus})}{\mathrm{d}t} &= -V\mathrm{\_Nucleus} \cdot ((\text{”k\_as\_Nrf2:Maf\_n\_(Nrf2\_ARE\_n)”} \cdot [\mathrm{Nrf2{:}Maf\_n}] \cdot [\mathrm{Nrf2\_ARE\_n}] - \mathrm{k\_ds\_Nrf2\_NMA\_n} \cdot [\mathrm{Nrf2\_NMA\_n}])) \\
\frac{\mathrm{d}([\mathrm{Nrf2\_DRE\_n}] \cdot V\mathrm{\_Nucleus})}{\mathrm{d}t} &= -V\mathrm{\_Nucleus} \cdot \Big(\text{”k\_as\_XNRT\_n\_(Nrf2\_DRE\_n)”} \cdot [\mathrm{Nrf2\_DRE\_n}] \cdot [\mathrm{XNRT\_n}]^{\text{”H\_as\_XNRT\_n\_(Nrf2\_DRE\_n)”}} \\
&\quad - \mathrm{k\_ds\_Nrf2\_XNRTD\_n} \cdot [\mathrm{Nrf2\_XNRTD\_n}]\Big)
\end{aligned}
$$

$$\frac{d([\text{Nrf2\_gene,a\_n}]\cdot V\text{\_Nucleus})}{dt} = +V\text{\_Nucleus}\cdot\Big(\text{k\_a\_b\_Nrf2\_gene, i\_n}\cdot[\text{Nrf2\_gene, i\_n}] + \text{"k\_a\_Nrf2\_gene, i\_n\_(Nrf2\_NMA\_n)"}\cdot[\text{Nrf2\_gene, i\_n}]\cdot[\text{Nrf2\_NMA\_n}]$$
$$+\text{"k\_a\_Nrf2\_gene, i\_n\_(Nrf2\_XNRTD\_n)"}\cdot[\text{Nrf2\_gene, i\_n}]\cdot[\text{Nrf2\_XNRTD\_n}] - \text{k\_i\_Nrf2\_gene, a\_n}\cdot[\text{Nrf2\_gene, a\_n}]\Big)$$

$$\frac{d([\text{Nrf2\_gene,i\_n}]\cdot V\text{\_Nucleus})}{dt} = -V\text{\_Nucleus}\cdot\Big(\text{k\_a\_b\_Nrf2\_gene, i\_n}\cdot[\text{Nrf2\_gene, i\_n}] + \text{"k\_a\_Nrf2\_gene, i\_n\_(Nrf2\_NMA\_n)"}\cdot[\text{Nrf2\_gene, i\_n}]\cdot[\text{Nrf2\_NMA\_n}]$$
$$+\text{"k\_a\_Nrf2\_gene, i\_n\_(Nrf2\_XNRTD\_n)"}\cdot[\text{Nrf2\_gene, i\_n}]\cdot[\text{Nrf2\_XNRTD\_n}] - \text{k\_i\_Nrf2\_gene, a\_n}\cdot[\text{Nrf2\_gene, a\_n}]\Big)$$

$$\frac{d([\text{Nrf2\_n}]\cdot V\text{\_Nucleus})}{dt} = -V\text{\_Nucleus}\cdot((\text{"k\_as\_Nrf2\_n\_(Maf\_n)"}\cdot[\text{Maf\_n}]\cdot[\text{Nrf2\_n}] - \text{k\_ds\_Nrf2:Maf\_n}\cdot[\text{Nrf2:Maf\_n}]))$$
$$+((\text{k\_tn\_Nrf2\_c}\cdot[\text{Nrf2\_c}] - \text{k\_tn\_Nrf2\_n}\cdot[\text{Nrf2\_n}])\cdot V\text{\_Nucleus})$$
$$-V\text{\_Nucleus}\cdot(\text{k\_d\_Nrf2\_n}\cdot[\text{Nrf2\_n}])$$

$$\frac{d([\text{Nrf2\_NMA\_n}]\cdot V\text{\_Nucleus})}{dt} = +V\text{\_Nucleus}\cdot((\text{"k\_as\_Nrf2:Maf\_n\_(Nrf2\_ARE\_n)"}\cdot[\text{Nrf2:Maf\_n}]\cdot[\text{Nrf2\_ARE\_n}] - \text{k\_ds\_Nrf2\_NMA\_n}\cdot[\text{Nrf2\_NMA\_n}]))$$

$$\frac{d([\text{Nrf2\_XNRTD\_n}]\cdot V\text{\_Nucleus})}{dt} = +V\text{\_Nucleus}\cdot\Big(\text{"k\_as\_XNRT\_n\_(Nrf2\_DRE\_n)"}\cdot[\text{Nrf2\_DRE\_n}]\cdot[\text{XNRT\_n}]^{\text{"H\_as\_XNRT\_n\_(Nrf2\_DRE\_n)"}}$$
$$-\text{k\_ds\_Nrf2\_XNRTD\_n}\cdot[\text{Nrf2\_XNRTD\_n}]\Big)$$

$$\frac{d([\text{NT\_n}]\cdot V\text{\_Nucleus})}{dt} = -V\text{\_Nucleus}\cdot((\text{"k\_as\_XNR\_n\_(NT\_n)"}\cdot[\text{XNR\_n}]\cdot[\text{NT\_n}] - \text{k\_ds\_XNRT\_n}\cdot[\text{XNRT\_n}]))$$

$$\frac{d([\text{X\_n}]\cdot V\text{\_Nucleus})}{dt} = +((\text{k\_df\_1\_X\_c}\cdot[\text{X\_c}] - \text{k\_df\_X\_n}\cdot[\text{X\_n}])\cdot V\text{\_Nucleus})$$
$$-V\text{\_Nucleus}\cdot((\text{"k\_as\_X\_n\_(NR\_n)"}\cdot[\text{NR\_n}]\cdot[\text{X\_n}] - \text{k\_ds\_XNR\_n}\cdot[\text{XNR\_n}]))$$

$$\frac{d([\text{XNR\_n}]\cdot V\text{\_Nucleus})}{dt} = +((\text{k\_tn\_XNR\_c}\cdot[\text{XNR\_c}] - \text{k\_tn\_XNR\_n}\cdot[\text{XNR\_n}])\cdot V\text{\_Nucleus})$$
$$+V\text{\_Nucleus}\cdot((\text{"k\_as\_X\_n\_(NR\_n)"}\cdot[\text{NR\_n}]\cdot[\text{X\_n}] - \text{k\_ds\_XNR\_n}\cdot[\text{XNR\_n}]))$$
$$-V\text{\_Nucleus}\cdot((\text{"k\_as\_XNR\_n\_(NT\_n)"}\cdot[\text{XNR\_n}]\cdot[\text{NT\_n}] - \text{k\_ds\_XNRT\_n}\cdot[\text{XNRT\_n}]))$$

$$
\begin{aligned}
\frac{\mathrm{d}([\text{XNRT\_n}]\cdot V\text{\_Nucleus})}{\mathrm{d}t} = & -V\text{\_Nucleus}\cdot((\text{"k\_as\_XNRT\_n\_(ABC3\_DRE\_n)"}\cdot[\text{XNRT\_n}]\cdot[\text{ABC3\_DRE\_n}]-\text{k\_ds\_ABC3\_XNRTD\_n}\cdot[\text{ABC3\_XNRTD\_n}]))\\
& -2\cdot V\text{\_Nucleus}\cdot\Big(\text{"k\_as\_XNRT\_n\_(CYP\_DRE\_n)"}\cdot[\text{CYP\_DRE\_n}]\cdot[\text{XNRT\_n}]^{\text{"H\_as\_XNRT\_n\_(CYP\_DRE\_n)"}}\\
& -\text{k\_ds\_CYP\_XNRTD\_n}\cdot[\text{CYP\_XNRTD\_n}]\Big)\\
& -2\cdot V\text{\_Nucleus}\cdot\Big(\text{"k\_as\_XNRT\_n\_(GST\_DRE\_n)"}\cdot[\text{GST\_DRE\_n}]\cdot[\text{XNRT\_n}]^{\text{"H\_as\_XNRT\_n\_(GST\_DRE\_n)"}}\\
& -\text{k\_ds\_GST\_XNRTD\_n}\cdot[\text{GST\_XNRTD\_n}]\Big)\\
& -2\cdot V\text{\_Nucleus}\cdot\Big(\text{"k\_as\_XNRT\_n\_(Nrf2\_DRE\_n)"}\cdot[\text{Nrf2\_DRE\_n}]\cdot[\text{XNRT\_n}]^{\text{"H\_as\_XNRT\_n\_(Nrf2\_DRE\_n)"}}\\
& -\text{k\_ds\_Nrf2\_XNRTD\_n}\cdot[\text{Nrf2\_XNRTD\_n}]\Big)\\
& -V\text{\_Nucleus}\cdot((\text{"k\_as\_XNRT\_n\_(ABC0\_DRE\_n)"}\cdot[\text{XNRT\_n}]\cdot[\text{ABC0\_DRE\_n}]-\text{k\_ds\_ABC0\_XNRTD\_n}\cdot[\text{ABC0\_XNRTD\_n}]))\\
& +V\text{\_Nucleus}\cdot((\text{"k\_as\_XNR\_n\_(NT\_n)"}\cdot[\text{XNR\_n}]\cdot[\text{NT\_n}]-\text{k\_ds\_XNRT\_n}\cdot[\text{XNRT\_n}]))
\end{aligned}
$$
