# Supplementary material for: A Novel Mathematical Model Describing Adaptive Cellular Drug Metabolism and Toxicity in the Chemoimmune System
Source: PLoS One. 2015 Feb 20;10(2):e0115533. doi: 10.1371/journal.pone.0115533 (PMC4338831; doi:10.1371/journal.pone.0115533)
Supplement: S3 Table — Parameters changed (compared to their default values shown in S2 Table) in simulation experiments. (PDF) [file pone.0115533.s007.pdf]

Figure	Panel	Curve	Parameter	Value
3	A-D	100x	k_df_1_X_c, k_df_2_X_c, k_df_X_e, k_df_X_n	10000
3	A-D	10x	k_df_1_X_c, k_df_2_X_c, k_df_X_e, k_df_X_n	1000
3	A-D	1x	k_df_1_X_c, k_df_2_X_c, k_df_X_e, k_df_X_n	100
3	A-D	0.1x	k_df_1_X_c, k_df_2_X_c, k_df_X_e, k_df_X_n	10
3	A-D	0.01x	k_df_1_X_c, k_df_2_X_c, k_df_X_e, k_df_X_n	1
3	E-H	100x	Km_tp_X_c_@ABC0_c	7000
3	E-H	10x	Km_tp_X_c_@ABC0_c	700
3	E-H	1x	Km_tp_X_c_@ABC0_c	70
3	E-H	0.1x	Km_tp_X_c_@ABC0_c	7
3	E-H	0.01x	Km_tp_X_c_@ABC0_c	0.7
4	all	100x	Km_cj_X'_c_@GST_c, Km_cj_X'b_c_@GST_c	5000000
4	all	10x	Km_cj_X'_c_@GST_c, Km_cj_X'b_c_@GST_c	500000
4	all	1x	Km_cj_X'_c_@GST_c, Km_cj_X'b_c_@GST_c	50000
4	all	0.1x	Km_cj_X'_c_@GST_c, Km_cj_X'b_c_@GST_c	5000
4	all	0.01x	Km_cj_X'_c_@GST_c, Km_cj_X'b_c_@GST_c	500
6	A	$[X_{c,crit}]$ = 0.05 nM	P_Toxicity_X_c	20
6	A	$[X_{c,crit}]$ = 0.5 nM	P_Toxicity_X_c	2
6	A	$[X_{c,crit}]$ = 5 nM	P_Toxicity_X_c	0.2
6	A	$[X_{c,crit}]$ = 5000 nM	P_Toxicity_X_c	0.0002
6	B	all	P_Toxicity_X_c	20
6	C	all	P_Toxicity_X_c	0.0002
6	B-C	0.2x ABC_0 transcription	k_ts_ABC0_gene,a_n	0.0018
6	B-C	5x *CYP* transcription	k_ts_CYP_gene,a_n	5.35
6	B-C	0.2x *CYP* transcription	k_ts_CYP_gene,a_n	0.214
6	B-C	5x ABC_0 transcription	k_ts_ABC0_gene,a_n	0.045
S2		all	P_Toxicity_X'_c, P_Toxicity_X'b_c	0.0002
S2		all	P_Toxicity_X''_c, P_Toxicity_X''b_c	2.00E-07
S3	all	$K_{m,ABC0}$ = 100x, rate of diffusion = 0.033x	Km_tp_X_c_@ABC0_c	7000
S3	all	$K_{m,ABC0}$ = 100x, rate of diffusion = 0.033x	k_df_1_X_c, k_df_2_X_c, k_df_X_e, k_df_X_n	3.3

S3	all	$K_{m,ABC0}$ = 10x, rate of diffusion = 0.13x	Km_tp_X_c_@ABC0_c	700
S3	all	$K_{m,ABC0}$ = 10x, rate of diffusion = 0.13x	k_df_1_X_c, k_df_2_X_c, k_df_X_e, k_df_X_n	13
S3	all	$K_{m,ABC0}$ = 1x, rate of diffusion = 1x	Km_tp_X_c_@ABC0_c	70
S3	all	$K_{m,ABC0}$ = 1x, rate of diffusion = 1x	k_df_1_X_c, k_df_2_X_c, k_df_X_e, k_df_X_n	100
S3	all	$K_{m,ABC0}$ = 0.1x, rate of diffusion = 7x	Km_tp_X_c_@ABC0_c	7
S3	all	$K_{m,ABC0}$ = 0.1x, rate of diffusion = 7x	k_df_1_X_c, k_df_2_X_c, k_df_X_e, k_df_X_n	700
S3	all	$K_{m,ABC0}$ = 0.01x, rate of diffusion = 20x	Km_tp_X_c_@ABC0_c	0.7
S3	all	$K_{m,ABC0}$ = 0.01x, rate of diffusion = 20x	k_df_1_X_c, k_df_2_X_c, k_df_X_e, k_df_X_n	2000
