# Supplementary material for: A Novel Mathematical Model Describing Adaptive Cellular Drug Metabolism and Toxicity in the Chemoimmune System
Source: PLoS One. 2015 Feb 20;10(2):e0115533. doi: 10.1371/journal.pone.0115533 (PMC4338831; doi:10.1371/journal.pone.0115533)
Supplement: S5 Table — SBML parameter types: F: fixed, A: assignment, O: ODE, B: Boolean (fixed with values 0 and 1, set by events), E: fixed, set by events. Min. and Max. values in parentheses indicate the actual values of minimums and maximums, respectively. For events see S6 Table. (PDF) [file pone.0115533.s009.pdf]

Variable	Ty-pe	Min.	Abbreviation, equation	Max.	Comment
			Chemical load calculation		
Baseline chemical load damage	F		$BCL_d = 0$		Baseline chemical load, damage part.
Baseline chemical load regeneration	F		$BCL_r = 0$		Baseline chemical load, regeneration part.
Maximal chemical load damage	F		$MCL_d = 99$		Maximal chemical load, damage part.
Maximal chemical load regeneration	F		$MCL_r = 1$		Maximal chemical load, regeneration part.
EC50 damage	F		$EC_{50d} = 222$		CLB which causes half maximal CL of the damage part.
EC50 regeneration	F		$EC_{50r} = 0.5$		CLB which causes half maximal CL of the regeneration part.
Hill coefficient of chemical load damage	F		$HCCL_d = 1.75$		Hill coefficient used for CL calculation, damage part.
Hill coefficient of chemical load regeneration	F		$HCCL_r = 7$		Hill coefficient used for CL calculation, regeneration part.
Toxicity i	F		$T_i = \text{variable}$		Toxicity of the ith toxic compound, expressed in inverse concentration units.
Chemical load base	A	0	$CLB = \sum_i^n (T_i \cdot X_i)$		Base (independent variable) of CL calculation.
Chemical load	A	$BCL_r + BCL_d$ (0)	$CL = BCL_r + \frac{MCL_r \cdot CLB^{HCCL_r}}{EC_{50r}^{HCCL_r} + CLB^{HCCL_r}} + BCL_d + \frac{MCL_d \cdot CLB^{HCCL_d}}{EC_{50d}^{HCCL_d} + CLB^{HCCL_d}}$	$MCL_r + MCL_d$ (100)	Overall toxic load caused by all of the toxic species.
			Damage and Regeneration calculation		
Regeneration capacity	F		$RC = 1$		Maximal CL which the cell can compensate without damage by its regeneration/repair mechanisms.
Damage	O		$D = -\int_{t_0=0}^{t} \frac{CL - RC + \lvert CL - RC \rvert}{2} dt$	0	Cumulative damage the cell bore since the start of the time course simulation experiment.
Potential regeneration	O	0	$PR = \int_{t_0=0}^{t} \frac{RC - CL + \lvert RC - CL \rvert}{2} dt$		Cumulative regeneration the cell potentially bore since the start of the time course simulation experiment.

Undamaged	B	0	$U = \begin{cases} 0 \text{ if } F < MF \text{ (from Reg. on)} \\ 1 \text{ if } F = MF \text{ (from Reg. off)} \end{cases}$	1	Indicates whether Fitness is maximal.
Regeneration threshold	E	0	$RT = PR$ (changes at Reg. off)		PR at the last Regeneration off event.
Unutilized regeneration helper	E	0	$URH = PR - RT + URH$ (changes at Reg. on)		UR at the last Regeneration on event.
Unutilized regeneration	A	0	$UR = U\frac{PR - RT + \lvert PR - RT \rvert}{2} + URH$		Cumulative regeneration the cell could not utilize (because of being undamaged) since the start of the time course simulation experiment.
Regeneration	A	0	$R = PR - UR$		Cumulative regeneration the cell bore since the start of the time course simulation experiment.
			Fitness calculation		
Fitness amplifier	F		$FA = 2 \cdot 10^{-5}$		Scaling factor applied in Fitness calculation. Determines how sensitive the cell is for Damage and Regeneration.
Maximal fitness	F		$MF = 1$		Maximal value of Fitness.
Fitness helper	A		$FH = FA\frac{D + R - \lvert D + R \rvert}{2} + MF$	*MF* (1)	Effect of Damage and Regeneration on Fitness.
Lethal fitness threshold	F		$LFT = 0$		Fitness threshold, where the cell dies.
Alive	B	0	$A = \begin{cases} 1 \text{ from } t_0 = 0\ h \\ 0 \text{ from Cell death} \end{cases}$	1	Indicates whether the cell is still alive or died due to toxication.
Fitness	A	*LFT* (0)	$F = A\frac{FH - LFT + \lvert FH - LFT \rvert}{2} + LFT$	*MF* (1)	Cellular fitness is the resultant of Damage, Regeneration and potential Cell death event in cell's history.
