# Supplementary material for: A Novel Mathematical Model Describing Adaptive Cellular Drug Metabolism and Toxicity in the Chemoimmune System
Source: PLoS One. 2015 Feb 20;10(2):e0115533. doi: 10.1371/journal.pone.0115533 (PMC4338831; doi:10.1371/journal.pone.0115533)
Supplement: S6 Table — For variable names see S5 Table. (PDF) [file pone.0115533.s010.pdf]

**Event**	**Trigger**	**Effect**	**Comment**
Cell death	$FH \leq LFT$	$A = 0$	FH reached LFT, the cell dies.
Regeneration on	$U(FH - MF) < -10^{-6}$	$U = 0$<br>$URH = URH + PR - RT$	The cell became damaged, regeneration may happen.
Regeneration off	$\lvert U - 1 \rvert (D + R) > 10^{-6}$	$U = 1$<br>$RT = PR$	Fitness became maximal, no more regeneration is possible.
