# Supplementary material for: A Novel Mathematical Model Describing Adaptive Cellular Drug Metabolism and Toxicity in the Chemoimmune System
Source: PLoS One. 2015 Feb 20;10(2):e0115533. doi: 10.1371/journal.pone.0115533 (PMC4338831; doi:10.1371/journal.pone.0115533)
Supplement: S1 Text — Details of cellular fitness, in silico cytotoxicity curve, and drug intake calculation. (PDF) [file pone.0115533.s011.pdf]

Supplementary methods

Details of cellular fitness, *in silico* cytotoxicity curve, and drug intake calculation are provided as follows.

Calculation of cellular fitness

The calculation of cellular fitness was implemented using SBML parameters and events listed in Table S5 and Table S6, respectively. It includes the definition and determination of `Chemical load`, `Damage` and `Regeneration`, and finally `Fitness` as listed below.

Calculation of `Chemical load`

The toxicity of intracellular toxic species (X_c, X_n, X'_c, X'_{bc}, X''_c and X''_{bc}) is stored in corresponding `Toxicity` (T_i) parameters, which are the reciprocals of their `Critical concentration`. The product of the concentration and the `Toxicity` of a given compound ($X_i \cdot T_i$) is proportional to the load which the given species influences on the cell. The sum of these products makes the `Chemical load base` (CLB), which is the independent variable of `Chemical load` (CL) calculation. The latter is calculated as the sum of two Hill equations, which are commonly used to simulate the relationship between drug effect and drug dose [1]. This way the `Chemical load` function has a shape of two 'stacked' sigmoidal curves, which results in more realistic *in silico* cytotoxicity curves, where the difference between NOAEL (no observed adverse effect level) and EC_{99} concentrations can be more than one order of magnitude. Parameters of the `Chemical load` function were determined in such a way, that $CL(CLB = 1) := 1$ (the value of `Regeneration capacity`), this way `Critical concentration` will be the threshold concentration which must be exceeded to cause cellular damage when the given compound is assumed to be the only toxic chemical in the cell. One of the Hill functions ('regeneration part') accounts mainly for `Chemical load` values lower than `Regeneration capacity`, while the other one ('damage part') accounts mainly for `Chemical load` values higher than `Regeneration capacity`.

Calculation of `Damage` and `Regeneration`

`Regeneration capacity` (RC) represents the cell's regeneration ability in response to chemical stress caused by toxic agents. It assumed to be constant and equals to the maximal `Chemical load` which the cell can compensate without damage. When `Chemical load` is over `Regeneration capacity`, damage occurs. In the opposite case regeneration may happen (only if `Fitness` is not maximal). To measure the overall damage which the cell bore from the start of the experiment (t_0) up to time `t`, `Damage` (D) is defined as the area under curve of the nonnegative part of the `(Chemical load – Regeneration capacity)` function, multiplied by -1 to have nonpositive values. (`Damage` has a value of zero at the beginning of the simulation, decreases when damage happens and stays constant otherwise.) Similarly, to measure the overall regeneration which the cell bore from the start of the experiment up to time `t`, `Regeneration` (R) is defined as the area under curve of the nonnegative part of the `(Regeneration capacity – Chemical load)` function, *considering only the area where `Fitness` is below its maximal value*. (`Regeneration` has a value of zero at the beginning of the simulation, grows when regeneration happens and stays constant otherwise.) To satisfy the latter criterion, `Regeneration` is calculated using several helper functions and two events as follows. The full (that is irrespective of `Fitness`) area under curve of the nonnegative part of the `(Regeneration capacity – Chemical load)` function was named `Potential Regeneration` (PR), since it represents the regeneration potential the cell may utilize when `Chemical load` is below `Regeneration capacity`. The `Undamaged` (U) variable indicates whether cell is damaged or undamaged. It is treated as Boolean: it is set to zero at the `Regeneration on` event (when `Fitness` drops below its maximal value, `Maximal fitness` (MF)) and is set to one at the `Regeneration off` event (when `Fitness` reaches `Maximal fitness`). `Regeneration threshold` (RT) stores the value of `Potential Regeneration` at the last `Regeneration off` event. `Unutilized regeneration helper` (URH) stores the value of `Unutilized regeneration` at the last `Regeneration on` event. `Unutilized regeneration` (UR) represents that part of `Potential Regeneration`, which could not be utilized, since the cell is undamaged (`Fitness` already reached `Maximal fitness`). It can be calculated by taking `Unutilized regeneration helper` and when `Fitness` is maximal (U = 1) and adding the nonnegative part of the difference of `Potential Regeneration` and

Regeneration threshold to that. Finally, the difference of Potential Regeneration and Unutilized regeneration gives Regeneration. This definition of Regeneration ensures that it never exceeds the absolute value of Damage which is a sane consideration in our case, when simulation experiments are started from a state of maximal Fitness.

Calculation of **Fitness**

The sum of Damage and Regeneration is indicative on cellular fitness. By adding its scaled (by the scaling factor Fitness amplifier (FA)) nonpositive value to Maximal fitness we get Fitness helper (FH). (Fitness amplifier is required to dampen the effect of the possibly high absolute values of Damage and Regeneration. It can be used to fine tune how sensitive the system is for a certain difference of Chemical load and Regeneration capacity.) When Fitness helper reaches the Lethal fitness threshold (LFT), event Cell death triggers, which sets the variable Alive (A) from its initial value one (indicating that the cell is alive) to zero (indicating that the cell is dead). Fitness (F) is defined as the sum of Lethal fitness threshold and the nonnegative part of the (FH - LFT) function multiplied by Alive. This definition ensures that Fitness is equal to Fitness helper till it is over the Lethal fitness threshold (that is before a Cell death event) and stays equal to Lethal fitness threshold afterwards.

Events

The Cell death event occurs when Fitness helper reaches the Lethal fitness threshold. It sets Alive to zero, which indicates the death of the cell. The Regeneration on and Regeneration off events are intended to monitor whether the cell became damaged or undamaged, respectively, and set the values of Undamaged, Unutilized regeneration helper and Regeneration threshold. Ideally, we should investigate *in both cases* whether Fitness is equal to Maximal fitness or not. Due to the way Fitness calculation is implemented, this would lead to cyclic dependencies. Also, comparison the equality of floating point variables could be problematic. Therefore we do not compare the value of $(FH - MF)$ to zero, but define a small threshold in both directions. The Regeneration on event triggers when FH drops below MF with more than one millionth of MF. Since FH never grows over MF, the Regeneration off

event triggers when Regeneration exceeds the absolute value of Damage with more than one millionth of MF. (This is the reason why the equation of Fitness helper contains not only the $(D + R)$ sum, but the nonpositive value of it.) Deviation from exact values applied in these two expressions certainly introduce some inaccuracy to the calculation, but it is negligible, since the applied threshold is very small. The utilization of variable Undamaged in trigger expressions of Regeneration on and Regeneration off events ensures that these events are triggered in a strictly alternating manner.

Calculation of in silico cytotoxicity curves

In silico cytotoxicity curves (Minimal Fitness *vs* [X_e] diagrams, Figure 6) were created in the following way. Critical concentrations of toxic species (X_c, X'_c and X''_c) were set to given values indicated on the figure. Time course simulations were run up to 48 hour as described in Methods using different extracellular drug concentrations ([X_e]). The minimal value of Fitness reached in each simulation was plotted against the corresponding [X_e] value (colored circles). By using minimal Fitness values in 48 hours instead of terminal Fitness values (observed at 48 hours), it is possible to differentiate between cases when Fitness did not decrease at all during the simulation experiment and cases when Fitness transiently decreased, but reached its maximal value before the end of the experiment. Additionally, Minimal Fitness *vs* X_e curves resemble more closely to experimentally determined cytotoxicity curves, since those reach the maximal Fitness value in smooth, 'almost asymptotic' fashion (with decreasing [X_e] values) instead of having a corner point which often would be the case when Fitness(48h) *vs* [X_e] curves were calculated. The continuous lines crossing the points described above are cubic B-spline interpolation curves and give an approximation of the minimal Fitness value between those points. This approximation is good enough for medium Fitness values (among others for the calculation of the EC_{50} value) but usually less reliable close to maximal and minimal Fitness values.

Calculation of drug intake (n(X_{in}))

The amount of cumulated drug intake in Figure 2 (n(X_{in})) is defined as the sum of the amount of all intracellular species containing some form of the drug, plus the amount of the ABC_{III}-excreted extracellular form of X'' (n(X''e)). It is calculated using SBML parameters.

$n(X_{in}) = n(X_c) + n(X_n) + n(X'_c) + n(X''_c) + n(XNR_c) + n(XNR_n) + n(XNRT_n) + n(ABC0\_XNRTD_n) + n(CYP\_XNRTD_n) + n(GST\_XNRTD_n) + n(ABC3\_XNRTD_n) + n(Nrf2\_XNRTD_n) + n(X''_e)$

References

1. Goutelle S, Maurin M, Rougier F, Barbaut X, Bourguignon L, et al. (2008) The Hill equation: a review of its capabilities in pharmacological modelling. Fundamental & clinical pharmacology 22: 633-648.
